# Ba_11_La_4_Br_34_: a new barium lanthanum bromide

**DOI:** 10.1107/S1600536811037354

**Published:** 2011-09-20

**Authors:** Yetta Eagleman, Guang Wu, Gautam Gundiah, Edith Bourret-Courchesne, Stephen Derenzo

**Affiliations:** a Lawrence Berkeley National Laboratory, One Cyclotron Rd, Berkeley, CA 94720, USA; b Department of Chemistry and Biochemistry, University of California Santa Barbara, Santa Barbara, CA 93106, USA

## Abstract

The structure of the title compound, barium lanthanum bromide (11/4/34), can be derived from the fluorite structure. The asymmetric unit contains two Ba sites (one with site symmetry 4/*m*..), one La site (site symmetry 4..), one mixed-occupied Ba and La site (ratio 1:1, site symmetry *m*..) and six Br sites (one with site symmetry \=4.., one with 2.., one with *m*.., the latter being disordered over two positions with a 0.86:0.14 ratio). The fundamental building units of the structure are edge-sharing polyhedral clusters made up of Ba and La bromide clusters inter­connected to BaBr_8_ square prisms and BaBr_10_ groups.

## Related literature

Alkaline earth halides (Cherepy *et al.*, 2008 ▶), rare earth halides (van Loef *et al.*, 2002 ▶; Glodo *et al.*, 2008 ▶), and compounds based on such binaries (Bourret-Courchesne *et al.*, 2009 ▶, 2010 ▶) are efficient scintillators when doped with divalent europium or trivalent cerium. For a detailed study of the luminescence properties of the title compound, see: Eagleman *et al.* (2011 ▶). Similar structure types to that of the title compound have been observed in ternary alkaline earth and rare earth fluorides (Bevan *et al.*, 1980 ▶, 1982 ▶; Burns *et al.*, 1968 ▶), chlorides (Liu & Eick, 1988 ▶, 1999 ▶; Löchner & Blachnik, 2011 ▶; Meyer & Masselmann, 1998 ▶), and bromides (Masselmann & Meyer, 1999 ▶; Liu & Eick, 1989 ▶) and in mixed valent rare earth halides (Druding & Corbett 1961 ▶; Liu & Eick, 1991 ▶). For structural details of simple and complex halides, see: Meyer & Wickleder (2000 ▶). For structural details of these types of superstructures, see: Meyer & Masselmann (1998 ▶). 

## Experimental

### 

#### Crystal data

Ba_11_La_4_Br_34_
*M* *_r_* = 4783.10
Tetragonal, 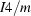

*a* = 11.909 (3) Å
*c* = 22.888 (5) Å
*V* = 3246.2 (10) Å^3^
*Z* = 2
Mo *K*α radiation
μ = 30.05 mm^−1^
*T* = 150 K
0.25 × 0.15 × 0.1 mm

#### Data collection

Bruker SMART1000 CCD area-detector diffractometer
Absorption correction: multi-scan (*SADABS*; Sheldrick, 1996 ▶) *T*_min_ = 0.141, *T*_max_ = 0.403
12169 measured reflections
1687 independent reflections
1193 reflections with *I* > 2σ(*I*)
*R*_int_ = 0.168

#### Refinement

*R*[*F* ^2^ > 2σ(*F* ^2^)] = 0.049
*wR*(*F* ^2^) = 0.118
*S* = 1.00
1687 reflections
63 parameters
(Δ/σ)_max_ = 0.100
Δρ_max_ = 5.72 e Å^−3^
Δρ_min_ = −3.31 e Å^−3^

### 

Data collection: *SMART* (Bruker, 2007 ▶); cell refinement: *SAINT* (Bruker, 2007 ▶); data reduction: *SAINT*; program(s) used to solve structure: *SHELXS97* (Sheldrick, 2008 ▶); program(s) used to refine structure: *SHELXL97* (Sheldrick, 2008 ▶); molecular graphics: *DIAMOND* (Brandenburg, 2005 ▶); software used to prepare material for publication: *publCIF* (Westrip, 2010 ▶).

## Supplementary Material

Crystal structure: contains datablock(s) I, global. DOI: 10.1107/S1600536811037354/ru2009sup1.cif

Structure factors: contains datablock(s) I. DOI: 10.1107/S1600536811037354/ru2009Isup2.hkl

Additional supplementary materials:  crystallographic information; 3D view; checkCIF report

## Figures and Tables

**Fig. 1.**
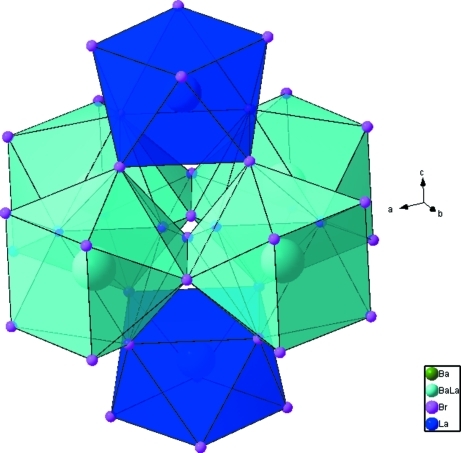
Representation of [{(Ba/La)6La4Br36}] polyhedral cluster

**Fig. 2.**
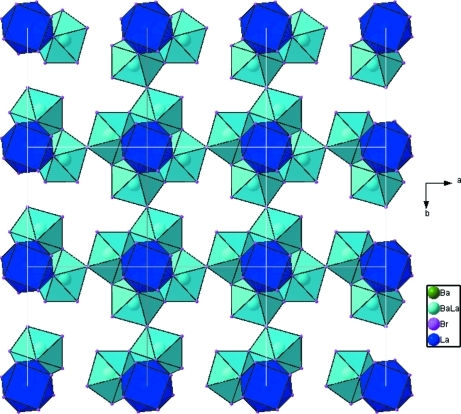
Connectivity of [{(Ba/La)6La4Br36}] polyhedral cluster along a-b plane

**Fig. 3.**
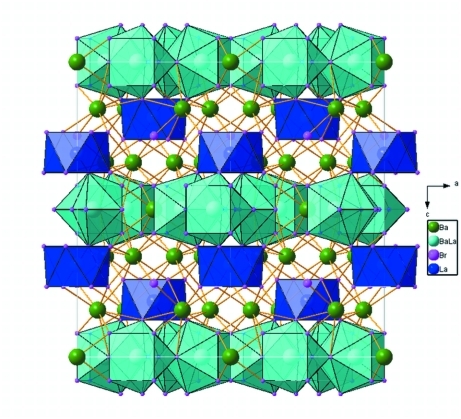
Structure of Ba11La4Br34viewing along (010) axis

## supplementary crystallographic information

### Comment

Alkaline earth halides (Cherepy *et al.* 2008), rare earth halides (van Loef *et al.* 2002; Glodo *et al.* 2008), and compounds based on such binaries (Bourret-Courchesne *et al.* 2010; Bourret-Courchesne *et al.* 2009) are efficient scintillators when doped with divalent europium or trivalent cerium. In an effort to discover new scintillators, a new mixed alkaline earth – rare earth bromide, Ba_11_La_4_Br_34_, has been obtained. When doped with either aforementioned activator this material displays high luminosities making them attractive as a promising scintillators. A detailed study of the luminescence properties will be presented in a future publication (Eagleman *et al.* 2011).

Ba_11_La_4_Br_34_ has a three dimensional tetragonal superstructure which can be derived from the fluorite structure. Similar structure types have been observed in ternary alkaline earth and rare earth fluorides (Bevan *et al.* 1980; Bevan *et al.* 1982; Burns *et al.* 1968), chlorides (Liu & Eick 1988; Liu & Eick 1999; Löchner & Blachnik 2011; Meyer & Masselmann 1998), and bromides (Masselmann & Meyer 1999; Liu & Eick 1989) and in mixed valent rare earth halides (Druding & Corbett 1961; Liu & Eick 1991). These superstructures follow the general formula of *M*_n_*X*_2n+5_ forming either a rhombohedral (n = 14) or tetragonal (n = 15) structure and consists of [*M*_6_Z*X*_36_] polyhedral clusters (Meyer & Wickleder 2000). There is some confusion about whether the interstitial atom, *Z*, is a halide or an oxide.

In Ba_11_La_4_Br_34_, n = 15 following the *M*_15_*X*_35_ = MZ*X*_34_ general formula. There are six bromine sites, two barium sites (Ba1 & Ba2), one site that is occupied by Ba(3) and La(1) atoms, and one lanthanum site (La2). Each Ba1 is coordinated to 8 bromines in square prism arrangements and have *4/m* site symmetry and Ba—Br distances of 3.2521 (12) Å. Each Ba(2) is coordinated to 10 bromines and have *1* symmetry and Ba—Br bond distances ranging from 3.2632 (12) – 3.7696 (13) Å. The Ba(3) and La(1) cations occupy the same site at 50% occupancy each. They are coordinated to 10 bromines and have *m* symmetry and bond distances ranging from 2.9708 (3) – 3.4805 (14) Å. Each La(2) is coordinated to 8 bromines in square antiprism arrangement and have *4* symmetry and La—Br distances of 3.0833 (15) Å (4x) and 3.1052 (15) Å (4x).

Typically, [*M*_6_Z*X*_36_] polyhedral clusters consist of six corner sharing *MX*_8_ square antiprisms whose metals are arranged in an octahedral geometry and the *Z* atom occupies the octahedral site. In the case of Ba_11_La_4_Br_34_, the clusters consist of four edge sharing Ba(3)/La(1)Br_10_ groups and capped by two La(2)Br_8_ square antiprisms having a [(Ba(3)/La(1))_4_La(2)_2_Br_16_Br_40/2_] formulation, shown in Figure 1. The interstitial atom, *Z*, is not present. The clusters are connected *via* four outer edges parallel to the (100) and (010) axis, Figure 2. The overall structure is made three dimensional by the interconnectivity of the clusters to Ba(1) and Ba(2) cations, Figure 3.

### Experimental

Small crystals of Ba_11_La_4_Br_34_ were formed from solid state reaction of a stoichiometric mixture of barium bromide and lanthanum bromide. The reactants were sealed in an evacuated quartz ampoule, heated at 1000 °C for 10 hr, and then slow cooled to room temperature at a rate of 0.5 °C/hr.

### Refinement

(type here to add refinement details)

### Crystal data

**Table:**

Ba_11_La_4_Br_34_	*D*_x_ = 4.894 Mg m^−^^3^
*M**_r_* = 4783.10	Mo *K*α radiation, λ = 0.71073 Å
Tetragonal, *I*4/*m*	Cell parameters from 119 reflections
*a* = 11.909 (3) Å	θ = 12.9–37.1°
*c* = 22.888 (5) Å	µ = 30.05 mm^−^^1^
*V* = 3246.2 (10) Å^3^	*T* = 150 K
*Z* = 2	Block, colorless
*F*(000) = 4068	0.25 × 0.15 × 0.1 mm

### Data collection

**Table:**

Bruker SMART1000 CCD area-detector diffractometer	1687 independent reflections
Radiation source: fine-focus sealed tube	1193 reflections with *I* > 2σ(*I*)
graphite	*R*_int_ = 0.168
ω scans	θ_max_ = 26.4°, θ_min_ = 1.9°
Absorption correction: multi-scan (*SADABS*; Sheldrick, 1996)	*h* = −14→14
*T*_min_ = 0.141, *T*_max_ = 0.403	*k* = −14→14
12169 measured reflections	*l* = −28→28

### Refinement

**Table:**

Refinement on *F*^2^	0 restraints
Least-squares matrix: full	Primary atom site location: structure-invariant direct methods
*R*[*F*^2^ > 2σ(*F*^2^)] = 0.049	Secondary atom site location: difference Fourier map
*wR*(*F*^2^) = 0.118	*w* = 1/[σ^2^(*F*_o_^2^) + (0.0501*P*)^2^] where *P* = (*F*_o_^2^ + 2*F*_c_^2^)/3
*S* = 1.00	(Δ/σ)_max_ = 0.100
1687 reflections	Δρ_max_ = 5.72 e Å^−^^3^
63 parameters	Δρ_min_ = −3.31 e Å^−^^3^

### Special details

**Table:**

Experimental. Disclaimer: This document was prepared as an account of work sponsored by the United States Government. While this document is believed to contain correct information, neither the United States Government nor any agency thereof, nor The Regents of the University of California, nor any of their employees, makes any warranty, express or implied, or assumes any legal responsibility for the accuracy, completeness, or usefulness of any information, apparatus, product, or process disclosed, or represents that its use would not infringe privately owned rights. Reference herein to any specific commercial product, process, or service by its trade name, trademark, manufacturer, or otherwise, does not necessarily constitute or imply its endorsement, recommendation, or favoring by the United States Government or any agency thereof, or The Regents of the University of California. The views and opinions of authors expressed herein do not necessarily state or reflect those of the United States Government or any agency thereof or The Regents of the University of California.
Geometry. All e.s.d.'s (except the e.s.d. in the dihedral angle between two l.s. planes) are estimated using the full covariance matrix. The cell e.s.d.'s are taken into account individually in the estimation of e.s.d.'s in distances, angles and torsion angles; correlations between e.s.d.'s in cell parameters are only used when they are defined by crystal symmetry. An approximate (isotropic) treatment of cell e.s.d.'s is used for estimating e.s.d.'s involving l.s. planes.
Refinement. Refinement of *F*^2^ against ALL reflections. The weighted *R*-factor *wR* and goodness of fit *S* are based on *F*^2^, conventional *R*-factors *R* are based on *F*, with *F* set to zero for negative *F*^2^. The threshold expression of *F*^2^ > σ(*F*^2^) is used only for calculating *R*-factors(gt) *etc*. and is not relevant to the choice of reflections for refinement. *R*-factors based on *F*^2^ are statistically about twice as large as those based on *F*, and *R*- factors based on ALL data will be even larger.

### Fractional atomic coordinates and isotropic or equivalent isotropic displacement parameters (Å^2^)

**Table:**

	*x*	*y*	*z*	*U*_iso_*/*U*_eq_	Occ. (<1)
Ba1	1.0000	1.0000	0.0000	0.0070 (5)	
Ba2	1.13364 (7)	0.68518 (6)	0.15892 (3)	0.0095 (2)	
Ba3	0.81444 (11)	0.60582 (10)	0.0000	0.0184 (3)	0.50
Br1	1.20905 (10)	0.91792 (11)	0.08083 (5)	0.0103 (3)	
Br2	1.0000	0.5000	0.2500	0.0113 (6)	
Br3	1.0000	0.5000	0.08013 (7)	0.0086 (4)	
Br4	0.90821 (10)	0.80123 (10)	0.23802 (5)	0.0100 (3)	
Br5	1.21627 (12)	1.07440 (11)	0.37484 (6)	0.0190 (4)	
Br6A	0.7919 (2)	0.3517 (2)	0.0000	0.0264 (6)	0.86
Br6B	0.5329 (13)	0.5572 (11)	0.0000	0.0190 (4)	0.14
La1	0.81444 (11)	0.60582 (10)	0.0000	0.0184 (3)	0.50
La2	1.0000	1.0000	0.31170 (6)	0.0063 (3)	

### Atomic displacement parameters (Å^2^)

**Table:**

	*U*^11^	*U*^22^	*U*^33^	*U*^12^	*U*^13^	*U*^23^
Ba1	0.0081 (7)	0.0081 (7)	0.0046 (10)	0.000	0.000	0.000
Ba2	0.0103 (4)	0.0073 (4)	0.0110 (4)	−0.0016 (3)	−0.0052 (3)	−0.0002 (3)
Ba3	0.0373 (8)	0.0135 (6)	0.0045 (6)	0.0125 (6)	0.000	0.000
Br1	0.0110 (6)	0.0108 (7)	0.0092 (7)	−0.0022 (5)	−0.0023 (5)	−0.0005 (5)
Br2	0.0113 (8)	0.0113 (8)	0.0114 (13)	0.000	0.000	0.000
Br3	0.0119 (9)	0.0056 (8)	0.0084 (9)	−0.0004 (7)	0.000	0.000
Br4	0.0084 (6)	0.0114 (7)	0.0101 (7)	0.0008 (5)	−0.0016 (5)	0.0013 (5)
Br5	0.0190 (7)	0.0145 (7)	0.0237 (8)	−0.0003 (6)	−0.0103 (6)	−0.0024 (6)
Br6A	0.0470 (16)	0.0229 (13)	0.0092 (12)	−0.0234 (12)	0.000	0.000
Br6B	0.0190 (7)	0.0145 (7)	0.0237 (8)	−0.0003 (6)	−0.0103 (6)	−0.0024 (6)
La1	0.0373 (8)	0.0135 (6)	0.0045 (6)	0.0125 (6)	0.000	0.000
La2	0.0048 (5)	0.0048 (5)	0.0093 (8)	0.000	0.000	0.000

### Geometric parameters (Å, °)

**Table:**

Ba1—Br1^i^	3.2521 (12)	Br1—Ba2^iii^	3.3737 (14)
Ba1—Br1^ii^	3.2521 (12)	Br2—Ba2^xvi^	3.4266 (8)
Ba1—Br1^iii^	3.2521 (12)	Br2—Ba2^viii^	3.4266 (9)
Ba1—Br1	3.2521 (13)	Br2—Ba2^xvii^	3.4266 (9)
Ba1—Br1^iv^	3.2521 (13)	Br3—La1^xi^	3.1361 (14)
Ba1—Br1^v^	3.2521 (13)	Br3—Ba3^xi^	3.1361 (14)
Ba1—Br1^vi^	3.2521 (13)	Br3—Ba2^xvi^	3.2633 (12)
Ba1—Br1^vii^	3.2521 (12)	Br4—La2	3.1052 (14)
Ba2—Br3	3.2633 (12)	Br4—Ba2^xvii^	3.2839 (15)
Ba2—Br4^viii^	3.2839 (15)	Br4—Ba2^ii^	3.3067 (15)
Ba2—Br4^iii^	3.3067 (14)	Br5—La2	3.0833 (15)
Ba2—Br1^ii^	3.3737 (15)	Br5—La1^xviii^	3.1167 (16)
Ba2—Br1	3.4182 (15)	Br5—Ba3^xviii^	3.1167 (16)
Ba2—Br2	3.4266 (8)	Br5—Ba2^xix^	3.5810 (16)
Ba2—Br4	3.5207 (15)	Br5—Ba2^x^	3.6536 (16)
Ba2—Br5^ix^	3.5810 (17)	Br6A—La1^xv^	2.971 (3)
Ba2—Br5^x^	3.6536 (16)	Br6A—Ba3^xv^	2.971 (3)
Ba2—Br6A^xi^	3.7696 (13)	Br6A—Ba2^xvi^	3.7696 (13)
Ba3—Br6A^xii^	2.971 (3)	Br6A—Ba2^xi^	3.7696 (13)
Ba3—Br6A	3.038 (3)	Br6B—Br6B^xii^	1.111 (18)
Ba3—Br5^xiii^	3.1167 (16)	Br6B—Br6B^xv^	1.111 (18)
Ba3—Br5^xiv^	3.1167 (16)	Br6B—Br6B^xx^	1.57 (3)
Ba3—Br1^ii^	3.1308 (16)	Br6B—La1^xii^	3.480 (14)
Ba3—Br1^i^	3.1308 (16)	Br6B—Ba3^xii^	3.480 (14)
Ba3—Br3	3.1361 (14)	La2—Br5^vi^	3.0833 (15)
Ba3—Br3^xi^	3.1361 (14)	La2—Br5^ii^	3.0833 (15)
Ba3—Br6B	3.402 (15)	La2—Br5^iii^	3.0833 (15)
Ba3—Br6B^xv^	3.480 (14)	La2—Br4^iii^	3.1052 (15)
Br1—La1^iii^	3.1308 (16)	La2—Br4^ii^	3.1052 (15)
Br1—Ba3^iii^	3.1308 (16)	La2—Br4^vi^	3.1052 (15)
			
Br1^i^—Ba1—Br1^ii^	69.34 (5)	Br3—Ba3—Br6B	128.76 (15)
Br1^i^—Ba1—Br1^iii^	180.00 (4)	Br3^xi^—Ba3—Br6B	128.76 (15)
Br1^ii^—Ba1—Br1^iii^	110.66 (5)	Br6A^xii^—Ba3—Br6B^xv^	76.6 (2)
Br1^i^—Ba1—Br1	108.88 (2)	Br6A—Ba3—Br6B^xv^	56.6 (2)
Br1^ii^—Ba1—Br1	71.12 (2)	Br5^xiii^—Ba3—Br6B^xv^	67.22 (4)
Br1^iii^—Ba1—Br1	71.12 (2)	Br5^xiv^—Ba3—Br6B^xv^	67.22 (4)
Br1^i^—Ba1—Br1^iv^	71.12 (2)	Br1^ii^—Ba3—Br6B^xv^	132.90 (14)
Br1^ii^—Ba1—Br1^iv^	108.88 (2)	Br1^i^—Ba3—Br6B^xv^	132.90 (14)
Br1^iii^—Ba1—Br1^iv^	108.88 (2)	Br3—Ba3—Br6B^xv^	115.43 (18)
Br1—Ba1—Br1^iv^	180.0	Br3^xi^—Ba3—Br6B^xv^	115.43 (18)
Br1^i^—Ba1—Br1^v^	71.12 (2)	Br6B—Ba3—Br6B^xv^	18.5 (3)
Br1^ii^—Ba1—Br1^v^	108.88 (2)	La1^iii^—Br1—Ba3^iii^	0.00 (4)
Br1^iii^—Ba1—Br1^v^	108.88 (2)	La1^iii^—Br1—Ba1	108.74 (4)
Br1—Ba1—Br1^v^	69.34 (5)	Ba3^iii^—Br1—Ba1	108.74 (4)
Br1^iv^—Ba1—Br1^v^	110.66 (5)	La1^iii^—Br1—Ba2^iii^	110.48 (4)
Br1^i^—Ba1—Br1^vi^	108.88 (2)	Ba3^iii^—Br1—Ba2^iii^	110.48 (4)
Br1^ii^—Ba1—Br1^vi^	71.12 (2)	Ba1—Br1—Ba2^iii^	111.00 (4)
Br1^iii^—Ba1—Br1^vi^	71.12 (2)	La1^iii^—Br1—Ba2	100.07 (4)
Br1—Ba1—Br1^vi^	110.66 (5)	Ba3^iii^—Br1—Ba2	100.07 (4)
Br1^iv^—Ba1—Br1^vi^	69.34 (5)	Ba1—Br1—Ba2	109.88 (4)
Br1^v^—Ba1—Br1^vi^	180.0	Ba2^iii^—Br1—Ba2	116.01 (4)
Br1^i^—Ba1—Br1^vii^	110.66 (5)	Ba2^xvi^—Br2—Ba2^viii^	111.721 (15)
Br1^ii^—Ba1—Br1^vii^	180.00 (4)	Ba2^xvi^—Br2—Ba2	105.06 (3)
Br1^iii^—Ba1—Br1^vii^	69.34 (5)	Ba2^viii^—Br2—Ba2	111.721 (18)
Br1—Ba1—Br1^vii^	108.88 (2)	Ba2^xvi^—Br2—Ba2^xvii^	111.721 (18)
Br1^iv^—Ba1—Br1^vii^	71.12 (2)	Ba2^viii^—Br2—Ba2^xvii^	105.06 (3)
Br1^v^—Ba1—Br1^vii^	71.12 (2)	Ba2—Br2—Ba2^xvii^	111.721 (15)
Br1^vi^—Ba1—Br1^vii^	108.88 (2)	Ba3—Br3—La1^xi^	108.43 (6)
Br3—Ba2—Br4^viii^	117.68 (4)	Ba3—Br3—Ba3^xi^	108.43 (6)
Br3—Ba2—Br4^iii^	163.70 (4)	La1^xi^—Br3—Ba3^xi^	0.00 (4)
Br4^viii^—Ba2—Br4^iii^	74.71 (4)	Ba3—Br3—Ba2	113.28 (2)
Br3—Ba2—Br1^ii^	65.68 (3)	La1^xi^—Br3—Ba2	104.54 (3)
Br4^viii^—Ba2—Br1^ii^	165.89 (4)	Ba3^xi^—Br3—Ba2	104.54 (3)
Br4^iii^—Ba2—Br1^ii^	99.72 (4)	Ba3—Br3—Ba2^xvi^	104.54 (3)
Br3—Ba2—Br1	112.77 (4)	La1^xi^—Br3—Ba2^xvi^	113.28 (2)
Br4^viii^—Ba2—Br1	119.38 (4)	Ba3^xi^—Br3—Ba2^xvi^	113.28 (2)
Br4^iii^—Ba2—Br1	64.74 (4)	Ba2—Br3—Ba2^xvi^	112.90 (6)
Br1^ii^—Ba2—Br1	67.68 (4)	La2—Br4—Ba2^xvii^	101.10 (4)
Br3—Ba2—Br2	71.02 (3)	La2—Br4—Ba2^ii^	113.66 (4)
Br4^viii^—Ba2—Br2	68.12 (3)	Ba2^xvii^—Br4—Ba2^ii^	105.29 (4)
Br4^iii^—Ba2—Br2	107.03 (3)	La2—Br4—Ba2	108.06 (4)
Br1^ii^—Ba2—Br2	102.06 (3)	Ba2^xvii^—Br4—Ba2	112.89 (4)
Br1—Ba2—Br2	164.16 (3)	Ba2^ii^—Br4—Ba2	115.03 (4)
Br3—Ba2—Br4	100.23 (3)	La2—Br5—La1^xviii^	141.14 (6)
Br4^viii^—Ba2—Br4	103.12 (3)	La2—Br5—Ba3^xviii^	141.14 (6)
Br4^iii^—Ba2—Br4	65.29 (4)	La1^xviii^—Br5—Ba3^xviii^	0.0
Br1^ii^—Ba2—Br4	62.95 (4)	La2—Br5—Ba2^xix^	95.25 (4)
Br1—Ba2—Br4	98.68 (4)	La1^xviii^—Br5—Ba2^xix^	96.92 (4)
Br2—Ba2—Br4	65.52 (3)	Ba3^xviii^—Br5—Ba2^xix^	96.92 (4)
Br3—Ba2—Br5^ix^	115.10 (4)	La2—Br5—Ba2^x^	93.81 (4)
Br4^viii^—Ba2—Br5^ix^	66.41 (4)	La1^xviii^—Br5—Ba2^x^	96.46 (4)
Br4^iii^—Ba2—Br5^ix^	78.89 (4)	Ba3^xviii^—Br5—Ba2^x^	96.46 (4)
Br1^ii^—Ba2—Br5^ix^	125.90 (4)	Ba2^xix^—Br5—Ba2^x^	145.74 (5)
Br1—Ba2—Br5^ix^	63.14 (3)	La1^xv^—Br6A—Ba3^xv^	0.00 (5)
Br2—Ba2—Br5^ix^	130.45 (3)	La1^xv^—Br6A—Ba3	136.81 (11)
Br4—Ba2—Br5^ix^	144.17 (4)	Ba3^xv^—Br6A—Ba3	136.81 (11)
Br3—Ba2—Br5^x^	63.25 (3)	La1^xv^—Br6A—Ba2^xvi^	95.61 (4)
Br4^viii^—Ba2—Br5^x^	64.40 (3)	Ba3^xv^—Br6A—Ba2^xvi^	95.61 (4)
Br4^iii^—Ba2—Br5^x^	132.90 (4)	Ba3—Br6A—Ba2^xvi^	95.48 (4)
Br1^ii^—Ba2—Br5^x^	125.20 (4)	La1^xv^—Br6A—Ba2^xi^	95.61 (4)
Br1—Ba2—Br5^x^	116.55 (4)	Ba3^xv^—Br6A—Ba2^xi^	95.61 (4)
Br2—Ba2—Br5^x^	79.13 (3)	Ba3—Br6A—Ba2^xi^	95.48 (4)
Br4—Ba2—Br5^x^	144.49 (4)	Ba2^xvi^—Br6A—Ba2^xi^	149.57 (9)
Br5^ix^—Ba2—Br5^x^	64.33 (5)	Br6B^xii^—Br6B—Br6B^xv^	90.000 (7)
Br3—Ba2—Br6A^xi^	60.17 (4)	Br6B^xii^—Br6B—Br6B^xx^	45.000 (3)
Br4^viii^—Ba2—Br6A^xi^	120.71 (6)	Br6B^xv^—Br6B—Br6B^xx^	45.000 (9)
Br4^iii^—Ba2—Br6A^xi^	124.53 (5)	Br6B^xii^—Br6B—Ba3	174.7 (13)
Br1^ii^—Ba2—Br6A^xi^	73.22 (5)	Br6B^xv^—Br6B—Ba3	84.7 (13)
Br1—Ba2—Br6A^xi^	61.85 (4)	Br6B^xx^—Br6B—Ba3	129.7 (13)
Br2—Ba2—Br6A^xi^	128.40 (4)	Br6B^xii^—Br6B—La1^xii^	76.8 (13)
Br4—Ba2—Br6A^xi^	136.16 (5)	Br6B^xv^—Br6B—La1^xii^	166.8 (13)
Br5^ix^—Ba2—Br6A^xi^	64.89 (5)	Br6B^xx^—Br6B—La1^xii^	121.8 (13)
Br5^x^—Ba2—Br6A^xi^	65.31 (5)	Ba3—Br6B—La1^xii^	108.5 (3)
Br6A^xii^—Ba3—Br6A	133.19 (11)	Br6B^xii^—Br6B—Ba3^xii^	76.8 (13)
Br6A^xii^—Ba3—Br5^xiii^	80.78 (4)	Br6B^xv^—Br6B—Ba3^xii^	166.8 (13)
Br6A—Ba3—Br5^xiii^	81.22 (4)	Br6B^xx^—Br6B—Ba3^xii^	121.8 (13)
Br6A^xii^—Ba3—Br5^xiv^	80.78 (4)	Ba3—Br6B—Ba3^xii^	108.5 (3)
Br6A—Ba3—Br5^xiv^	81.22 (4)	La1^xii^—Br6B—Ba3^xii^	0.00 (3)
Br5^xiii^—Ba3—Br5^xiv^	133.61 (7)	Br5—La2—Br5^vi^	124.10 (7)
Br6A^xii^—Ba3—Br1^ii^	74.74 (6)	Br5—La2—Br5^ii^	77.31 (3)
Br6A—Ba3—Br1^ii^	137.40 (4)	Br5^vi^—La2—Br5^ii^	77.31 (3)
Br5^xiii^—Ba3—Br1^ii^	140.84 (5)	Br5—La2—Br5^iii^	77.31 (3)
Br5^xiv^—Ba3—Br1^ii^	71.89 (4)	Br5^vi^—La2—Br5^iii^	77.31 (3)
Br6A^xii^—Ba3—Br1^i^	74.74 (6)	Br5^ii^—La2—Br5^iii^	124.10 (7)
Br6A—Ba3—Br1^i^	137.40 (4)	Br5—La2—Br4	140.14 (3)
Br5^xiii^—Ba3—Br1^i^	71.89 (4)	Br5^vi^—La2—Br4	75.01 (4)
Br5^xiv^—Ba3—Br1^i^	140.84 (5)	Br5^ii^—La2—Br4	73.67 (4)
Br1^ii^—Ba3—Br1^i^	72.44 (5)	Br5^iii^—La2—Br4	142.20 (4)
Br6A^xii^—Ba3—Br3	140.27 (4)	Br5—La2—Br4^iii^	73.67 (4)
Br6A—Ba3—Br3	70.23 (5)	Br5^vi^—La2—Br4^iii^	142.20 (3)
Br5^xiii^—Ba3—Br3	138.90 (5)	Br5^ii^—La2—Br4^iii^	75.01 (4)
Br5^xiv^—Ba3—Br3	71.25 (4)	Br5^iii^—La2—Br4^iii^	140.14 (4)
Br1^ii^—Ba3—Br3	70.13 (3)	Br4—La2—Br4^iii^	72.85 (3)
Br1^i^—Ba3—Br3	110.56 (5)	Br5—La2—Br4^ii^	142.20 (3)
Br6A^xii^—Ba3—Br3^xi^	140.27 (4)	Br5^vi^—La2—Br4^ii^	73.67 (4)
Br6A—Ba3—Br3^xi^	70.23 (5)	Br5^ii^—La2—Br4^ii^	140.14 (4)
Br5^xiii^—Ba3—Br3^xi^	71.25 (4)	Br5^iii^—La2—Br4^ii^	75.01 (4)
Br5^xiv^—Ba3—Br3^xi^	138.90 (5)	Br4—La2—Br4^ii^	72.85 (3)
Br1^ii^—Ba3—Br3^xi^	110.56 (5)	Br4^iii^—La2—Br4^ii^	114.21 (6)
Br1^i^—Ba3—Br3^xi^	70.13 (3)	Br5—La2—Br4^vi^	75.01 (4)
Br3—Ba3—Br3^xi^	71.57 (6)	Br5^vi^—La2—Br4^vi^	140.14 (3)
Br6A^xii^—Ba3—Br6B	58.0 (2)	Br5^ii^—La2—Br4^vi^	142.20 (4)
Br6A—Ba3—Br6B	75.1 (2)	Br5^iii^—La2—Br4^vi^	73.67 (4)
Br5^xiii^—Ba3—Br6B	67.04 (4)	Br4—La2—Br4^vi^	114.21 (6)
Br5^xiv^—Ba3—Br6B	67.04 (4)	Br4^iii^—La2—Br4^vi^	72.85 (3)
Br1^ii^—Ba3—Br6B	120.51 (17)	Br4^ii^—La2—Br4^vi^	72.85 (3)
Br1^i^—Ba3—Br6B	120.51 (17)		
			
Br1^i^—Ba1—Br1—La1^iii^	53.75 (5)	Br5^ix^—Ba2—Br3—Ba2^xvi^	126.62 (4)
Br1^ii^—Ba1—Br1—La1^iii^	113.02 (6)	Br5^x^—Ba2—Br3—Ba2^xvi^	87.13 (3)
Br1^iii^—Ba1—Br1—La1^iii^	−126.25 (5)	Br6A^xi^—Ba2—Br3—Ba2^xvi^	162.64 (5)
Br1^iv^—Ba1—Br1—La1^iii^	−41 (100)	Br3—Ba2—Br4—La2	−174.93 (4)
Br1^v^—Ba1—Br1—La1^iii^	−6.61 (5)	Br4^viii^—Ba2—Br4—La2	63.28 (6)
Br1^vi^—Ba1—Br1—La1^iii^	173.39 (5)	Br4^iii^—Ba2—Br4—La2	−2.80 (5)
Br1^vii^—Ba1—Br1—La1^iii^	−66.98 (6)	Br1^ii^—Ba2—Br4—La2	−119.13 (5)
Br1^i^—Ba1—Br1—Ba3^iii^	53.75 (5)	Br1—Ba2—Br4—La2	−59.75 (5)
Br1^ii^—Ba1—Br1—Ba3^iii^	113.02 (6)	Br2—Ba2—Br4—La2	121.51 (4)
Br1^iii^—Ba1—Br1—Ba3^iii^	−126.25 (5)	Br5^ix^—Ba2—Br4—La2	−4.45 (8)
Br1^iv^—Ba1—Br1—Ba3^iii^	−41 (100)	Br5^x^—Ba2—Br4—La2	127.37 (6)
Br1^v^—Ba1—Br1—Ba3^iii^	−6.61 (5)	Br6A^xi^—Ba2—Br4—La2	−117.74 (7)
Br1^vi^—Ba1—Br1—Ba3^iii^	173.39 (5)	Br3—Ba2—Br4—Ba2^xvii^	74.12 (5)
Br1^vii^—Ba1—Br1—Ba3^iii^	−66.98 (6)	Br4^viii^—Ba2—Br4—Ba2^xvii^	−47.66 (4)
Br1^i^—Ba1—Br1—Ba2^iii^	175.49 (4)	Br4^iii^—Ba2—Br4—Ba2^xvii^	−113.75 (4)
Br1^ii^—Ba1—Br1—Ba2^iii^	−125.24 (2)	Br1^ii^—Ba2—Br4—Ba2^xvii^	129.92 (5)
Br1^iii^—Ba1—Br1—Ba2^iii^	−4.51 (4)	Br1—Ba2—Br4—Ba2^xvii^	−170.70 (4)
Br1^iv^—Ba1—Br1—Ba2^iii^	81 (100)	Br2—Ba2—Br4—Ba2^xvii^	10.56 (3)
Br1^v^—Ba1—Br1—Ba2^iii^	115.12 (3)	Br5^ix^—Ba2—Br4—Ba2^xvii^	−115.39 (6)
Br1^vi^—Ba1—Br1—Ba2^iii^	−64.88 (3)	Br5^x^—Ba2—Br4—Ba2^xvii^	16.43 (8)
Br1^vii^—Ba1—Br1—Ba2^iii^	54.76 (2)	Br6A^xi^—Ba2—Br4—Ba2^xvii^	131.31 (6)
Br1^i^—Ba1—Br1—Ba2	−54.85 (2)	Br3—Ba2—Br4—Ba2^ii^	−46.75 (5)
Br1^ii^—Ba1—Br1—Ba2	4.42 (4)	Br4^viii^—Ba2—Br4—Ba2^ii^	−168.54 (4)
Br1^iii^—Ba1—Br1—Ba2	125.15 (2)	Br4^iii^—Ba2—Br4—Ba2^ii^	125.38 (3)
Br1^iv^—Ba1—Br1—Ba2	−150 (100)	Br1^ii^—Ba2—Br4—Ba2^ii^	9.05 (4)
Br1^v^—Ba1—Br1—Ba2	−115.21 (3)	Br1—Ba2—Br4—Ba2^ii^	68.43 (4)
Br1^vi^—Ba1—Br1—Ba2	64.79 (3)	Br2—Ba2—Br4—Ba2^ii^	−110.31 (4)
Br1^vii^—Ba1—Br1—Ba2	−175.58 (4)	Br5^ix^—Ba2—Br4—Ba2^ii^	123.73 (5)
Br3—Ba2—Br1—La1^iii^	−69.57 (4)	Br5^x^—Ba2—Br4—Ba2^ii^	−104.45 (7)
Br4^viii^—Ba2—Br1—La1^iii^	74.96 (5)	Br6A^xi^—Ba2—Br4—Ba2^ii^	10.44 (8)
Br4^iii^—Ba2—Br1—La1^iii^	128.07 (4)	Br6A^xii^—Ba3—Br6A—La1^xv^	0.0
Br1^ii^—Ba2—Br1—La1^iii^	−118.64 (4)	Br5^xiii^—Ba3—Br6A—La1^xv^	−68.45 (3)
Br2—Ba2—Br1—La1^iii^	−170.37 (10)	Br5^xiv^—Ba3—Br6A—La1^xv^	68.45 (3)
Br4—Ba2—Br1—La1^iii^	−174.58 (4)	Br1^ii^—Ba3—Br6A—La1^xv^	119.19 (8)
Br5^ix^—Ba2—Br1—La1^iii^	38.07 (4)	Br1^i^—Ba3—Br6A—La1^xv^	−119.19 (8)
Br5^x^—Ba2—Br1—La1^iii^	0.80 (5)	Br3—Ba3—Br6A—La1^xv^	141.58 (3)
Br6A^xi^—Ba2—Br1—La1^iii^	−36.35 (5)	Br3^xi^—Ba3—Br6A—La1^xv^	−141.58 (3)
Br3—Ba2—Br1—Ba3^iii^	−69.57 (4)	Br6B—Ba3—Br6A—La1^xv^	0.0
Br4^viii^—Ba2—Br1—Ba3^iii^	74.96 (5)	Br6B^xv^—Ba3—Br6A—La1^xv^	0.0
Br4^iii^—Ba2—Br1—Ba3^iii^	128.07 (4)	Br6A^xii^—Ba3—Br6A—Ba3^xv^	0.0
Br1^ii^—Ba2—Br1—Ba3^iii^	−118.64 (4)	Br5^xiii^—Ba3—Br6A—Ba3^xv^	−68.45 (3)
Br2—Ba2—Br1—Ba3^iii^	−170.37 (10)	Br5^xiv^—Ba3—Br6A—Ba3^xv^	68.45 (3)
Br4—Ba2—Br1—Ba3^iii^	−174.58 (4)	Br1^ii^—Ba3—Br6A—Ba3^xv^	119.19 (8)
Br5^ix^—Ba2—Br1—Ba3^iii^	38.07 (4)	Br1^i^—Ba3—Br6A—Ba3^xv^	−119.19 (8)
Br5^x^—Ba2—Br1—Ba3^iii^	0.80 (5)	Br3—Ba3—Br6A—Ba3^xv^	141.58 (3)
Br6A^xi^—Ba2—Br1—Ba3^iii^	−36.35 (5)	Br3^xi^—Ba3—Br6A—Ba3^xv^	−141.58 (3)
Br3—Ba2—Br1—Ba1	44.70 (5)	Br6B—Ba3—Br6A—Ba3^xv^	0.0
Br4^viii^—Ba2—Br1—Ba1	−170.76 (4)	Br6B^xv^—Ba3—Br6A—Ba3^xv^	0.0
Br4^iii^—Ba2—Br1—Ba1	−117.65 (4)	Br6A^xii^—Ba3—Br6A—Ba2^xvi^	−104.22 (5)
Br1^ii^—Ba2—Br1—Ba1	−4.36 (4)	Br5^xiii^—Ba3—Br6A—Ba2^xvi^	−172.66 (6)
Br2—Ba2—Br1—Ba1	−56.10 (13)	Br5^xiv^—Ba3—Br6A—Ba2^xvi^	−35.77 (5)
Br4—Ba2—Br1—Ba1	−60.30 (4)	Br1^ii^—Ba3—Br6A—Ba2^xvi^	14.97 (12)
Br5^ix^—Ba2—Br1—Ba1	152.34 (5)	Br1^i^—Ba3—Br6A—Ba2^xvi^	136.60 (6)
Br5^x^—Ba2—Br1—Ba1	115.08 (4)	Br3—Ba3—Br6A—Ba2^xvi^	37.37 (5)
Br6A^xi^—Ba2—Br1—Ba1	77.92 (6)	Br3^xi^—Ba3—Br6A—Ba2^xvi^	114.20 (7)
Br3—Ba2—Br1—Ba2^iii^	171.61 (3)	Br6B—Ba3—Br6A—Ba2^xvi^	−104.22 (5)
Br4^viii^—Ba2—Br1—Ba2^iii^	−43.86 (6)	Br6B^xv^—Ba3—Br6A—Ba2^xvi^	−104.22 (5)
Br4^iii^—Ba2—Br1—Ba2^iii^	9.25 (4)	Br6A^xii^—Ba3—Br6A—Ba2^xi^	104.22 (5)
Br1^ii^—Ba2—Br1—Ba2^iii^	122.54 (3)	Br5^xiii^—Ba3—Br6A—Ba2^xi^	35.77 (5)
Br2—Ba2—Br1—Ba2^iii^	70.81 (12)	Br5^xiv^—Ba3—Br6A—Ba2^xi^	172.66 (6)
Br4—Ba2—Br1—Ba2^iii^	66.60 (4)	Br1^ii^—Ba3—Br6A—Ba2^xi^	−136.60 (6)
Br5^ix^—Ba2—Br1—Ba2^iii^	−80.75 (5)	Br1^i^—Ba3—Br6A—Ba2^xi^	−14.97 (12)
Br5^x^—Ba2—Br1—Ba2^iii^	−118.02 (5)	Br3—Ba3—Br6A—Ba2^xi^	−114.20 (7)
Br6A^xi^—Ba2—Br1—Ba2^iii^	−155.17 (7)	Br3^xi^—Ba3—Br6A—Ba2^xi^	−37.37 (5)
Br3—Ba2—Br2—Ba2^xvi^	0.0	Br6B—Ba3—Br6A—Ba2^xi^	104.22 (5)
Br4^viii^—Ba2—Br2—Ba2^xvi^	−131.87 (3)	Br6B^xv^—Ba3—Br6A—Ba2^xi^	104.22 (5)
Br4^iii^—Ba2—Br2—Ba2^xvi^	162.98 (4)	Br6A^xii^—Ba3—Br6B—Br6B^xii^	180.0
Br1^ii^—Ba2—Br2—Ba2^xvi^	58.74 (3)	Br6A—Ba3—Br6B—Br6B^xii^	0.0
Br1—Ba2—Br2—Ba2^xvi^	106.71 (12)	Br5^xiii^—Ba3—Br6B—Br6B^xii^	86.61 (10)
Br4—Ba2—Br2—Ba2^xvi^	111.28 (3)	Br5^xiv^—Ba3—Br6B—Br6B^xii^	−86.61 (10)
Br5^ix^—Ba2—Br2—Ba2^xvi^	−107.23 (4)	Br1^ii^—Ba3—Br6B—Br6B^xii^	−136.69 (10)
Br5^x^—Ba2—Br2—Ba2^xvi^	−65.25 (3)	Br1^i^—Ba3—Br6B—Br6B^xii^	136.69 (10)
Br6A^xi^—Ba2—Br2—Ba2^xvi^	−19.29 (6)	Br3—Ba3—Br6B—Br6B^xii^	−48.58 (14)
Br3—Ba2—Br2—Ba2^viii^	121.314 (8)	Br3^xi^—Ba3—Br6B—Br6B^xii^	48.58 (14)
Br4^viii^—Ba2—Br2—Ba2^viii^	−10.56 (3)	Br6B^xv^—Ba3—Br6B—Br6B^xii^	0.0
Br4^iii^—Ba2—Br2—Ba2^viii^	−75.71 (4)	Br6A^xii^—Ba3—Br6B—Br6B^xv^	180.0
Br1^ii^—Ba2—Br2—Ba2^viii^	−179.94 (3)	Br6A—Ba3—Br6B—Br6B^xv^	0.0
Br1—Ba2—Br2—Ba2^viii^	−131.98 (12)	Br5^xiii^—Ba3—Br6B—Br6B^xv^	86.61 (10)
Br4—Ba2—Br2—Ba2^viii^	−127.41 (3)	Br5^xiv^—Ba3—Br6B—Br6B^xv^	−86.61 (10)
Br5^ix^—Ba2—Br2—Ba2^viii^	14.08 (4)	Br1^ii^—Ba3—Br6B—Br6B^xv^	−136.69 (10)
Br5^x^—Ba2—Br2—Ba2^viii^	56.06 (2)	Br1^i^—Ba3—Br6B—Br6B^xv^	136.69 (10)
Br6A^xi^—Ba2—Br2—Ba2^viii^	102.03 (6)	Br3—Ba3—Br6B—Br6B^xv^	−48.58 (14)
Br3—Ba2—Br2—Ba2^xvii^	−121.314 (12)	Br3^xi^—Ba3—Br6B—Br6B^xv^	48.58 (14)
Br4^viii^—Ba2—Br2—Ba2^xvii^	106.82 (3)	Br6A^xii^—Ba3—Br6B—Br6B^xx^	180.0
Br4^iii^—Ba2—Br2—Ba2^xvii^	41.67 (4)	Br6A—Ba3—Br6B—Br6B^xx^	0.0
Br1^ii^—Ba2—Br2—Ba2^xvii^	−62.57 (2)	Br5^xiii^—Ba3—Br6B—Br6B^xx^	86.61 (10)
Br1—Ba2—Br2—Ba2^xvii^	−14.60 (11)	Br5^xiv^—Ba3—Br6B—Br6B^xx^	−86.61 (10)
Br4—Ba2—Br2—Ba2^xvii^	−10.03 (3)	Br1^ii^—Ba3—Br6B—Br6B^xx^	−136.69 (10)
Br5^ix^—Ba2—Br2—Ba2^xvii^	131.46 (5)	Br1^i^—Ba3—Br6B—Br6B^xx^	136.69 (10)
Br5^x^—Ba2—Br2—Ba2^xvii^	173.43 (3)	Br3—Ba3—Br6B—Br6B^xx^	−48.58 (14)
Br6A^xi^—Ba2—Br2—Ba2^xvii^	−140.60 (6)	Br3^xi^—Ba3—Br6B—Br6B^xx^	48.58 (14)
Br6A^xii^—Ba3—Br3—La1^xi^	−150.15 (11)	Br6B^xv^—Ba3—Br6B—Br6B^xx^	0.0
Br6A—Ba3—Br3—La1^xi^	74.99 (4)	Br6A^xii^—Ba3—Br6B—La1^xii^	0.0
Br5^xiii^—Ba3—Br3—La1^xi^	26.19 (6)	Br6A—Ba3—Br6B—La1^xii^	180.0
Br5^xiv^—Ba3—Br3—La1^xi^	162.16 (4)	Br5^xiii^—Ba3—Br6B—La1^xii^	−93.39 (10)
Br1^ii^—Ba3—Br3—La1^xi^	−120.93 (4)	Br5^xiv^—Ba3—Br6B—La1^xii^	93.39 (10)
Br1^i^—Ba3—Br3—La1^xi^	−59.49 (3)	Br1^ii^—Ba3—Br6B—La1^xii^	43.31 (10)
Br3^xi^—Ba3—Br3—La1^xi^	0.0	Br1^i^—Ba3—Br6B—La1^xii^	−43.31 (10)
Br6B—Ba3—Br3—La1^xi^	125.4 (2)	Br3—Ba3—Br6B—La1^xii^	131.42 (14)
Br6B^xv^—Ba3—Br3—La1^xi^	110.05 (17)	Br3^xi^—Ba3—Br6B—La1^xii^	−131.42 (14)
Br6A^xii^—Ba3—Br3—Ba3^xi^	−150.15 (11)	Br6B^xv^—Ba3—Br6B—La1^xii^	180.0
Br6A—Ba3—Br3—Ba3^xi^	74.99 (4)	Br6A^xii^—Ba3—Br6B—Ba3^xii^	0.0
Br5^xiii^—Ba3—Br3—Ba3^xi^	26.19 (6)	Br6A—Ba3—Br6B—Ba3^xii^	180.0
Br5^xiv^—Ba3—Br3—Ba3^xi^	162.16 (4)	Br5^xiii^—Ba3—Br6B—Ba3^xii^	−93.39 (10)
Br1^ii^—Ba3—Br3—Ba3^xi^	−120.93 (4)	Br5^xiv^—Ba3—Br6B—Ba3^xii^	93.39 (10)
Br1^i^—Ba3—Br3—Ba3^xi^	−59.49 (3)	Br1^ii^—Ba3—Br6B—Ba3^xii^	43.31 (10)
Br3^xi^—Ba3—Br3—Ba3^xi^	0.0	Br1^i^—Ba3—Br6B—Ba3^xii^	−43.31 (10)
Br6B—Ba3—Br3—Ba3^xi^	125.4 (2)	Br3—Ba3—Br6B—Ba3^xii^	131.42 (14)
Br6B^xv^—Ba3—Br3—Ba3^xi^	110.05 (17)	Br3^xi^—Ba3—Br6B—Ba3^xii^	−131.42 (14)
Br6A^xii^—Ba3—Br3—Ba2	−34.59 (12)	Br6B^xv^—Ba3—Br6B—Ba3^xii^	180.0
Br6A—Ba3—Br3—Ba2	−169.45 (6)	La1^xviii^—Br5—La2—Br5^vi^	0.77 (6)
Br5^xiii^—Ba3—Br3—Ba2	141.75 (7)	Ba3^xviii^—Br5—La2—Br5^vi^	0.77 (6)
Br5^xiv^—Ba3—Br3—Ba2	−82.28 (5)	Ba2^xix^—Br5—La2—Br5^vi^	−107.10 (4)
Br1^ii^—Ba3—Br3—Ba2	−5.37 (4)	Ba2^x^—Br5—La2—Br5^vi^	105.98 (3)
Br1^i^—Ba3—Br3—Ba2	56.07 (6)	La1^xviii^—Br5—La2—Br5^ii^	−64.11 (7)
Br3^xi^—Ba3—Br3—Ba2	115.56 (4)	Ba3^xviii^—Br5—La2—Br5^ii^	−64.11 (7)
Br6B—Ba3—Br3—Ba2	−119.1 (2)	Ba2^xix^—Br5—La2—Br5^ii^	−171.99 (3)
Br6B^xv^—Ba3—Br3—Ba2	−134.39 (17)	Ba2^x^—Br5—La2—Br5^ii^	41.09 (5)
Br6A^xii^—Ba3—Br3—Ba2^xvi^	88.72 (10)	La1^xviii^—Br5—La2—Br5^iii^	65.66 (7)
Br6A—Ba3—Br3—Ba2^xvi^	−46.14 (4)	Ba3^xviii^—Br5—La2—Br5^iii^	65.66 (7)
Br5^xiii^—Ba3—Br3—Ba2^xvi^	−94.93 (8)	Ba2^xix^—Br5—La2—Br5^iii^	−42.21 (6)
Br5^xiv^—Ba3—Br3—Ba2^xvi^	41.04 (4)	Ba2^x^—Br5—La2—Br5^iii^	170.87 (3)
Br1^ii^—Ba3—Br3—Ba2^xvi^	117.95 (5)	La1^xviii^—Br5—La2—Br4	−108.12 (10)
Br1^i^—Ba3—Br3—Ba2^xvi^	179.38 (4)	Ba3^xviii^—Br5—La2—Br4	−108.12 (10)
Br3^xi^—Ba3—Br3—Ba2^xvi^	−121.12 (3)	Ba2^xix^—Br5—La2—Br4	144.01 (7)
Br6B—Ba3—Br3—Ba2^xvi^	4.2 (2)	Ba2^x^—Br5—La2—Br4	−2.91 (9)
Br6B^xv^—Ba3—Br3—Ba2^xvi^	−11.07 (17)	La1^xviii^—Br5—La2—Br4^iii^	−141.98 (8)
Br4^viii^—Ba2—Br3—Ba3	169.88 (4)	Ba3^xviii^—Br5—La2—Br4^iii^	−141.98 (8)
Br4^iii^—Ba2—Br3—Ba3	32.77 (17)	Ba2^xix^—Br5—La2—Br4^iii^	110.15 (4)
Br1^ii^—Ba2—Br3—Ba3	5.14 (4)	Ba2^x^—Br5—La2—Br4^iii^	−36.77 (4)
Br1—Ba2—Br3—Ba3	−44.94 (5)	La1^xviii^—Br5—La2—Br4^ii^	109.38 (11)
Br2—Ba2—Br3—Ba3	118.59 (4)	Ba3^xviii^—Br5—La2—Br4^ii^	109.38 (11)
Br4—Ba2—Br3—Ba3	59.07 (5)	Ba2^xix^—Br5—La2—Br4^ii^	1.51 (10)
Br5^ix^—Ba2—Br3—Ba3	−114.79 (4)	Ba2^x^—Br5—La2—Br4^ii^	−145.42 (8)
Br5^x^—Ba2—Br3—Ba3	−154.28 (6)	La1^xviii^—Br5—La2—Br4^vi^	141.89 (9)
Br6A^xi^—Ba2—Br3—Ba3	−78.77 (7)	Ba3^xviii^—Br5—La2—Br4^vi^	141.89 (9)
Br4^viii^—Ba2—Br3—La1^xi^	−72.27 (6)	Ba2^xix^—Br5—La2—Br4^vi^	34.02 (4)
Br4^iii^—Ba2—Br3—La1^xi^	150.61 (13)	Ba2^x^—Br5—La2—Br4^vi^	−112.90 (4)
Br1^ii^—Ba2—Br3—La1^xi^	122.99 (5)	Ba2^xvii^—Br4—La2—Br5	87.56 (9)
Br1—Ba2—Br3—La1^xi^	72.91 (4)	Ba2^ii^—Br4—La2—Br5	−160.15 (7)
Br2—Ba2—Br3—La1^xi^	−123.56 (3)	Ba2—Br4—La2—Br5	−31.19 (10)
Br4—Ba2—Br3—La1^xi^	176.92 (4)	Ba2^xvii^—Br4—La2—Br5^vi^	−38.25 (4)
Br5^ix^—Ba2—Br3—La1^xi^	3.06 (5)	Ba2^ii^—Br4—La2—Br5^vi^	74.05 (5)
Br5^x^—Ba2—Br3—La1^xi^	−36.43 (3)	Ba2—Br4—La2—Br5^vi^	−157.00 (5)
Br6A^xi^—Ba2—Br3—La1^xi^	39.07 (6)	Ba2^xvii^—Br4—La2—Br5^ii^	42.63 (4)
Br4^viii^—Ba2—Br3—Ba3^xi^	−72.27 (6)	Ba2^ii^—Br4—La2—Br5^ii^	154.93 (5)
Br4^iii^—Ba2—Br3—Ba3^xi^	150.61 (13)	Ba2—Br4—La2—Br5^ii^	−76.12 (4)
Br1^ii^—Ba2—Br3—Ba3^xi^	122.99 (5)	Ba2^xvii^—Br4—La2—Br5^iii^	−82.52 (9)
Br1—Ba2—Br3—Ba3^xi^	72.91 (4)	Ba2^ii^—Br4—La2—Br5^iii^	29.78 (11)
Br2—Ba2—Br3—Ba3^xi^	−123.56 (3)	Ba2—Br4—La2—Br5^iii^	158.74 (8)
Br4—Ba2—Br3—Ba3^xi^	176.92 (4)	Ba2^xvii^—Br4—La2—Br4^iii^	121.58 (5)
Br5^ix^—Ba2—Br3—Ba3^xi^	3.06 (5)	Ba2^ii^—Br4—La2—Br4^iii^	−126.12 (2)
Br5^x^—Ba2—Br3—Ba3^xi^	−36.43 (3)	Ba2—Br4—La2—Br4^iii^	2.83 (5)
Br6A^xi^—Ba2—Br3—Ba3^xi^	39.07 (6)	Ba2^xvii^—Br4—La2—Br4^ii^	−115.43 (5)
Br4^viii^—Ba2—Br3—Ba2^xvi^	51.29 (3)	Ba2^ii^—Br4—La2—Br4^ii^	−3.13 (5)
Br4^iii^—Ba2—Br3—Ba2^xvi^	−85.82 (14)	Ba2—Br4—La2—Br4^ii^	125.82 (2)
Br1^ii^—Ba2—Br3—Ba2^xvi^	−113.45 (3)	Ba2^xvii^—Br4—La2—Br4^vi^	−176.92 (4)
Br1—Ba2—Br3—Ba2^xvi^	−163.52 (4)	Ba2^ii^—Br4—La2—Br4^vi^	−64.63 (4)
Br2—Ba2—Br3—Ba2^xvi^	0.0	Ba2—Br4—La2—Br4^vi^	64.33 (3)
Br4—Ba2—Br3—Ba2^xvi^	−59.51 (2)		

Symmetry codes: (i) *y*, −*x*+2, −*z*; (ii) *y*, −*x*+2, *z*; (iii) −*y*+2, *x*, *z*; (iv) −*x*+2, −*y*+2, −*z*; (v) *x*, *y*, −*z*; (vi) −*x*+2, −*y*+2, *z*; (vii) −*y*+2, *x*, −*z*; (viii) *y*+1/2, −*x*+3/2, −*z*+1/2; (ix) −*y*+5/2, *x*−1/2, −*z*+1/2; (x) −*x*+5/2, −*y*+3/2, −*z*+1/2; (xi) −*x*+2, −*y*+1, −*z*; (xii) −*y*+1, *x*, *z*; (xiii) *x*−1/2, *y*−1/2, *z*−1/2; (xiv) *x*−1/2, *y*−1/2, −*z*+1/2; (xv) *y*, −*x*+1, −*z*; (xvi) −*x*+2, −*y*+1, *z*; (xvii) −*y*+3/2, *x*−1/2, −*z*+1/2; (xviii) *x*+1/2, *y*+1/2, *z*+1/2; (xix) *y*+1/2, −*x*+5/2, −*z*+1/2; (xx) −*x*+1, −*y*+1, −*z*.

### Table 1 Observed and calculated structure factors of BA11La4 Br34

**Table:**

h	k	l	Fo	Fc	s	h	k	l	Fo	Fc	s	h	k	l	Fo	Fc	s	h	k	l	Fo	Fc	s	h	k	l	Fo	Fc	s	
1	1	0	76	74	4	2	3	1	272	273	7	4	4	2	77	68	7	-2	5	3	309	311	5	0	6	4	131	127	6	
0	2	0	99	89	7	-3	4	1	158	138	4	-3	5	2	155	165	4	0	5	3	1091	1095	25	2	6	4	105	113	7	
2	2	0	115	85	8	1	4	1	717	693	24	-1	5	2	69	69	7	2	5	3	515	524	10	4	6	4	203	185	4	
1	3	0	26	10	25	3	4	1	49	46	11	1	5	2	322	306	8	4	5	3	380	368	7	6	6	4	291	281	6	
3	3	0	917	852	22	-4	5	1	44	29	15	3	5	2	130	150	5	-5	6	3	259	255	7	-5	7	4	49	80	22	
0	4	0	43	54	16	-2	5	1	203	194	4	5	5	2	269	266	5	-3	6	3	36	21	24	-3	7	4	55	64	14	
4	4	0	283	285	5	0	5	1	150	141	4	-4	6	2	196	188	5	-1	6	3	78	81	10	-1	7	4	416	406	12	
-3	5	0	523	504	10	2	5	1	81	70	7	-2	6	2	253	240	5	1	6	3	185	186	6	1	7	4	93	105	8	
-1	5	0	29	10	28	4	5	1	82	93	8	0	6	2	59	94	13	3	6	3	894	862	10	3	7	4	106	109	8	
1	5	0	163	160	8	-5	6	1	106	110	7	2	6	2	150	159	5	5	6	3	591	538	9	5	7	4	287	288	9	
3	5	0	250	231	7	-3	6	1	520	505	9	4	6	2	117	115	7	-6	7	3	365	358	7	7	7	4	198	202	7	
5	5	0	401	391	9	-1	6	1	87	97	7	6	6	2	62	56	14	-4	7	3	597	573	9	-6	8	4	331	347	9	
-4	6	0	321	333	5	1	6	1	81	93	7	-5	7	2	99	89	9	-2	7	3	629	619	15	-4	8	4	356	345	7	
-2	6	0	1526	1646	40	3	6	1	283	267	4	-3	7	2	241	242	5	0	7	3	506	470	8	-2	8	4	57	58	20	
0	6	0	414	387	11	5	6	1	84	114	9	-1	7	2	140	149	6	2	7	3	345	331	5	0	8	4	124	134	10	
2	6	0	208	199	9	-6	7	1	210	209	6	1	7	2	281	276	5	4	7	3	34	5	34	2	8	4	239	232	6	
4	6	0	190	191	7	-4	7	1	218	202	5	3	7	2	156	141	5	-7	8	3	350	333	10	6	8	4	120	119	10	
6	6	0	488	474	5	-2	7	1	67	70	10	5	7	2	33	5	33	-5	8	3	320	306	9	8	8	4	184	191	12	
-5	7	0	29	13	29	0	7	1	48	15	19	7	7	2	0	42	1	-3	8	3	227	218	7	-7	9	4	70	80	26	
-3	7	0	252	260	9	2	7	1	21	16	21	-6	8	2	83	69	13	-1	8	3	595	558	10	-5	9	4	399	398	8	
-1	7	0	879	818	18	4	7	1	67	74	13	-4	8	2	237	228	6	1	8	3	647	648	11	-3	9	4	131	135	9	
1	7	0	326	335	11	6	7	1	39	47	39	-2	8	2	118	117	9	3	8	3	435	424	4	-1	9	4	322	304	6	
3	7	0	555	523	11	-7	8	1	189	184	10	0	8	2	0	3	1	5	8	3	37	7	37	1	9	4	101	96	11	
5	7	0	840	771	18	-5	8	1	56	69	20	2	8	2	126	119	9	7	8	3	406	380	17	3	9	4	189	193	8	
7	7	0	829	775	35	-3	8	1	358	351	9	4	8	2	26	16	25	-8	9	3	235	230	14	5	9	4	62	68	26	
-6	8	0	1008	980	27	-1	8	1	235	214	5	6	8	2	62	50	23	-6	9	3	170	185	9	7	9	4	36	40	36	
-4	8	0	437	446	18	1	8	1	299	301	6	8	8	2	72	76	22	-4	9	3	228	229	7	9	9	4	236	211	14	
-2	8	0	464	468	24	5	8	1	82	86	14	-7	9	2	366	353	11	-2	9	3	293	278	6	-8	10	4	182	173	12	
0	8	0	74	87	20	7	8	1	291	267	14	-5	9	2	26	17	25	0	9	3	47	73	28	-6	10	4	328	319	7	
2	8	0	152	178	12	-8	9	1	170	154	11	-3	9	2	313	310	7	2	9	3	543	537	9	-4	10	4	140	146	11	
4	8	0	1162	1078	33	-6	9	1	0	15	1	-1	9	2	344	314	6	4	9	3	314	296	7	-2	10	4	93	102	16	
6	8	0	616	559	42	-4	9	1	180	189	8	1	9	2	104	113	12	6	9	3	207	204	14	0	10	4	274	284	7	
8	8	0	39	25	38	-2	9	1	61	80	23	3	9	2	90	90	14	8	9	3	50	15	49	2	10	4	285	298	8	
-7	9	0	126	147	18	0	9	1	77	82	16	5	9	2	329	300	11	-9	10	3	472	431	25	4	10	4	215	218	8	
-5	9	0	807	778	12	2	9	1	60	45	23	7	9	2	216	214	15	-7	10	3	246	248	10	6	10	4	55	8	55	
-3	9	0	95	106	21	4	9	1	165	162	9	9	9	2	16	6	15	-5	10	3	45	50	44	8	10	4	45	74	44	
-1	9	0	613	594	16	6	9	1	45	15	45	-8	10	2	131	109	19	-3	10	3	706	652	9	10	10	4	308	278	33	
1	9	0	116	125	15	8	9	1	0	2	1	-6	10	2	79	92	23	-1	10	3	112	120	14	-9	11	4	0	12	1	
3	9	0	78	122	25	-7	10	1	49	45	48	-4	10	2	42	49	41	1	10	3	144	161	10	-7	11	4	59	79	58	
5	9	0	72	77	32	-5	10	1	308	312	7	-2	10	2	34	18	33	3	10	3	174	164	9	-5	11	4	181	186	11	
7	9	0	387	335	42	-3	10	1	187	198	9	0	10	2	74	81	19	5	10	3	358	326	13	-3	11	4	99	107	17	
9	9	0	0	46	1	-1	10	1	473	459	9	2	10	2	81	84	17	7	10	3	425	363	28	-1	11	4	426	418	9	
-8	10	0	149	176	26	1	10	1	159	168	9	4	10	2	45	18	45	9	10	3	351	309	22	1	11	4	102	94	15	
-6	10	0	254	237	12	3	10	1	106	111	13	6	10	2	0	23	1	-6	11	3	347	322	14	3	11	4	48	31	48	
-4	10	0	168	189	14	5	10	1	78	89	20	8	10	2	40	49	40	-4	11	3	141	160	13	5	11	4	155	148	12	
-2	10	0	48	13	48	7	10	1	42	47	41	10	10	2	99	83	39	-2	11	3	80	44	20	7	11	4	114	111	20	
0	10	0	1063	1038	22	9	10	1	293	271	31	-9	11	2	0	5	1	0	11	3	561	557	14	9	11	4	115	127	26	
2	10	0	906	848	31	-10	11	1	79	52	79	-7	11	2	284	278	17	2	11	3	88	87	18	-8	12	4	306	287	15	
4	10	0	53	44	53	-8	11	1	225	231	17	-5	11	2	210	214	10	4	11	3	106	76	16	-6	12	4	0	13	1	
6	10	0	88	18	34	-6	11	1	0	48	1	-3	11	2	47	31	46	6	11	3	338	314	22	-4	12	4	292	301	9	
8	10	0	426	391	54	-4	11	1	97	111	18	-1	11	2	0	32	1	8	11	3	165	163	21	-2	12	4	122	135	18	
10	10	0	552	457	57	-2	11	1	329	328	7	1	11	2	302	290	7	-7	12	3	311	312	13	0	12	4	108	111	16	
-9	11	0	233	221	24	0	11	1	44	85	44	3	11	2	199	210	9	-5	12	3	226	233	10	2	12	4	290	288	8	
-7	11	0	341	305	25	2	11	1	154	153	10	5	11	2	54	53	54	-3	12	3	178	189	12	4	12	4	228	219	9	
-5	11	0	510	501	9	4	11	1	134	118	12	7	11	2	19	15	18	-1	12	3	156	160	11	6	12	4	227	208	13	
-3	11	0	128	131	20	6	11	1	482	413	30	9	11	2	0	13	1	1	12	3	256	264	8	8	12	4	42	10	41	
-1	11	0	28	28	28	8	11	1	244	241	22	-8	12	2	0	47	1	3	12	3	372	365	8	-5	13	4	0	24	1	
1	11	0	310	301	16	10	11	1	237	159	48	-6	12	2	48	47	47	5	12	3	76	57	29	-3	13	4	0	5	1	
3	11	0	317	310	10	-7	12	1	86	104	37	-4	12	2	29	47	29	7	12	3	82	50	32	-1	13	4	0	15	1	
5	11	0	678	613	43	-5	12	1	145	158	15	-2	12	2	219	215	11	-6	13	3	47	55	46	1	13	4	175	185	13	
7	11	0	455	418	62	-3	12	1	60	97	43	0	12	2	0	13	1	-4	13	3	428	442	8	3	13	4	287	264	10	
9	11	0	260	222	31	-1	12	1	54	47	54	2	12	2	125	136	14	-2	13	3	215	216	12	5	13	4	209	180	26	
-8	12	0	538	476	34	1	12	1	153	142	11	4	12	2	89	85	21	0	13	3	88	95	22	-4	14	4	0	24	1	
-6	12	0	94	106	37	3	12	1	123	129	14	6	12	2	55	43	54	2	13	3	64	27	39	-2	14	4	48	62	48	
-4	12	0	791	758	11	5	12	1	206	208	14	8	12	2	52	16	51	4	13	3	183	189	12	0	14	4	221	224	12	
-2	12	0	345	331	17	7	12	1	97	70	26	-7	13	2	0	15	1	6	13	3	292	230	21	2	14	4	253	257	11	
0	12	0	200	208	14	-6	13	1	209	214	14	-5	13	2	115	153	22	-3	14	3	68	34	67	4	14	4	119	105	33	
2	12	0	251	255	13	-4	13	1	307	290	9	-3	13	2	150	165	15	-1	14	3	535	550	10	-1	2	5	182	167	8	
4	12	0	406	388	26	-2	13	1	75	99	32	-1	13	2	228	239	10	1	14	3	120	100	19	1	2	5	242	227	3	
6	12	0	918	788	86	0	13	1	60	42	41	1	13	2	177	184	11	3	14	3	107	103	23	-2	3	5	144	150	3	
8	12	0	423	369	51	2	13	1	61	19	43	3	13	2	99	69	20	1	1	4	94	84	3	0	3	5	186	175	5	
-7	13	0	76	79	76	4	13	1	179	185	12	5	13	2	25	21	24	0	2	4	168	170	9	2	3	5	594	578	12	
-5	13	0	354	337	19	6	13	1	82	53	34	7	13	2	298	241	25	2	2	4	299	279	6	-3	4	5	202	191	3	
-3	13	0	371	380	12	-5	14	1	39	74	38	-4	14	2	7	21	6	-1	3	4	423	417	9	-1	4	5	36	14	17	
-1	13	0	247	275	13	-3	14	1	209	228	14	-2	14	2	71	94	43	1	3	4	652	633	12	1	4	5	270	287	6	
1	13	0	520	541	13	-1	14	1	236	240	10	0	14	2	48	10	47	3	3	4	276	284	4	3	4	5	44	64	12	
3	13	0	295	297	12	1	14	1	120	142	17	2	14	2	122	109	19	-2	4	4	280	293	4	-4	5	5	527	493	10	
5	13	0	29	26	29	3	14	1	119	118	19	4	14	2	78	20	78	0	4	4	30	8	21	-2	5	5	76	82	6	
7	13	0	33	55	33	5	14	1	72	119	71	0	1	3	168	181	6	2	4	4	412	397	8	0	5	5	182	173	7	
-4	14	0	324	328	26	1	1	2	168	172	4	-1	2	3	55	73	4	4	4	4	52	70	11	2	5	5	108	108	6	
-2	14	0	0	7	1	0	2	2	46	38	5	-2	3	3	228	221	3	-3	5	4	0	14	1	4	5	5	182	173	5	
0	14	0	485	483	12	2	2	2	0	11	1	0	3	3	114	108	3	-1	5	4	98	95	6	-5	6	5	341	321	9	
2	14	0	473	467	19	-1	3	2	296	263	4	-3	4	3	1131	1116	18	1	5	4	103	97	5	-3	6	5	223	230	4	
4	14	0	554	546	24	1	3	2	59	40	6	-1	4	3	600	593	12	3	5	4	81	107	8	-1	6	5	138	145	8	
0	1	1	33	5	5	-2	4	2	105	79	6	1	4	3	377	386	8	5	5	4	131	134	6	1	6	5	139	140	5	
-1	2	1	21	8	20	0	4	2	19	24	18	3	4	3	411	380	6	-4	6	4	616	589	13	3	6	5	226	233	5	
0	3	1	149	133	5	2	4	2	131	124	4	-4	5	3	179	157	4	-2	6	4	174	197	4	5	6	5	172	149	6	
-6	7	5	85	94	13	7	7	6	158	154	8	-3	8	7	194	219	7	4	8	8	18	5	18	-2	9	9	53	52	31	
-4	7	5	159	149	6	-6	8	6	364	337	12	-1	8	7	332	325	5	6	8	8	46	33	46	0	9	9	122	137	11	
-2	7	5	96	102	8	-4	8	6	494	488	9	1	8	7	78	96	13	8	8	8	99	100	20	2	9	9	221	231	7	
0	7	5	183	169	5	-2	8	6	147	142	9	3	8	7	137	136	8	-7	9	8	97	123	22	4	9	9	634	605	13	
2	7	5	32	12	31	0	8	6	157	156	8	5	8	7	89	90	13	-5	9	8	152	159	12	6	9	9	312	310	17	
4	7	5	266	248	6	2	8	6	433	421	6	7	8	7	291	263	16	-3	9	8	188	190	9	8	9	9	0	27	1	
6	7	5	109	117	10	4	8	6	127	142	9	-8	9	7	396	378	22	-1	9	8	228	209	7	-9	10	9	274	276	15	
-7	8	5	47	2	47	6	8	6	197	200	11	-6	9	7	73	80	27	1	9	8	211	209	7	-7	10	9	284	289	19	
-5	8	5	0	23	1	8	8	6	654	583	31	-4	9	7	510	513	8	3	9	8	145	158	10	-5	10	9	169	193	12	
-3	8	5	82	105	13	-7	9	6	345	335	16	-2	9	7	0	5	1	5	9	8	117	115	13	-3	10	9	367	361	9	
-1	8	5	122	108	8	-5	9	6	22	23	21	0	9	7	0	14	1	7	9	8	125	107	14	-1	10	9	99	103	16	
1	8	5	121	126	9	-3	9	6	1009	961	17	2	9	7	21	11	21	9	9	8	61	31	60	1	10	9	108	121	13	
3	8	5	233	235	6	-1	9	6	392	402	5	4	9	7	186	189	8	-8	10	8	28	7	27	3	10	9	26	12	26	
5	8	5	170	172	7	1	9	6	101	111	12	6	9	7	760	653	43	-6	10	8	132	171	15	5	10	9	177	168	12	
7	8	5	200	180	9	3	9	6	76	94	17	8	9	7	141	139	15	-4	10	8	255	256	8	7	10	9	225	224	15	
-8	9	5	204	182	10	5	9	6	461	419	18	-9	10	7	100	128	27	-2	10	8	367	356	6	9	10	9	102	107	33	
-6	9	5	249	245	7	7	9	6	792	725	41	-7	10	7	387	383	14	0	10	8	162	178	9	-8	11	9	113	100	24	
-4	9	5	173	175	8	9	9	6	379	376	17	-5	10	7	353	371	10	2	10	8	131	123	11	-6	11	9	254	259	16	
-2	9	5	68	73	17	-8	10	6	326	328	17	-3	10	7	22	15	22	4	10	8	133	135	13	-4	11	9	191	212	10	
0	9	5	74	83	16	-6	10	6	52	58	51	-1	10	7	27	26	27	6	10	8	28	17	28	-2	11	9	251	258	8	
2	9	5	85	86	14	-4	10	6	111	126	14	1	10	7	0	20	1	8	10	8	388	378	30	0	11	9	595	596	6	
4	9	5	328	305	6	-2	10	6	599	581	7	3	10	7	42	32	42	10	10	8	27	65	27	2	11	9	241	255	9	
6	9	5	199	174	10	0	10	6	333	327	6	5	10	7	139	153	13	-9	11	8	58	101	57	4	11	9	87	97	22	
8	9	5	294	276	18	2	10	6	578	551	9	7	10	7	402	339	32	-7	11	8	23	8	22	6	11	9	0	42	1	
-9	10	5	15	18	14	4	10	6	529	494	15	9	10	7	71	60	71	-5	11	8	180	172	14	8	11	9	179	173	20	
-7	10	5	0	19	1	6	10	6	188	182	12	-8	11	7	217	215	14	-3	11	8	0	64	1	-7	12	9	105	143	52	
-5	10	5	64	80	26	8	10	6	224	191	16	-6	11	7	63	67	62	-1	11	8	108	134	19	-5	12	9	105	123	26	
-3	10	5	81	103	18	10	10	6	316	292	34	-4	11	7	28	11	28	1	11	8	0	20	1	-3	12	9	126	127	17	
-1	10	5	212	219	9	-9	11	6	126	116	51	-2	11	7	376	373	7	3	11	8	114	99	16	-1	12	9	110	118	19	
1	10	5	117	138	12	-7	11	6	493	485	14	0	11	7	288	272	8	5	11	8	206	208	11	1	12	9	217	211	10	
3	10	5	130	130	12	-5	11	6	223	232	12	2	11	7	542	539	9	7	11	8	72	69	47	3	12	9	333	338	14	
5	10	5	115	116	14	-3	11	6	225	233	10	4	11	7	257	235	13	9	11	8	0	47	1	5	12	9	155	156	17	
7	10	5	0	13	1	-1	11	6	75	74	29	6	11	7	357	306	21	-6	12	8	202	209	15	-4	13	9	234	262	13	
9	10	5	293	225	21	1	11	6	152	173	12	8	11	7	109	135	49	-4	12	8	338	339	9	-2	13	9	50	52	49	
-8	11	5	132	129	24	3	11	6	789	753	16	-7	12	7	0	22	1	-2	12	8	199	223	13	0	13	9	126	159	18	
-6	11	5	202	213	13	5	11	6	584	537	35	-5	12	7	193	206	14	0	12	8	79	68	26	2	13	9	105	103	22	
-4	11	5	82	86	24	7	11	6	0	12	1	-3	12	7	98	105	22	2	12	8	167	180	12	4	13	9	139	166	17	
-2	11	5	70	78	28	9	11	6	97	64	78	-1	12	7	166	182	12	4	12	8	318	314	13	-1	14	9	212	201	27	
0	11	5	111	122	14	-8	12	6	211	220	33	1	12	7	368	361	8	6	12	8	254	212	22	1	14	9	117	43	117	
2	11	5	106	123	15	-6	12	6	484	498	12	3	12	7	112	98	18	-5	13	8	143	110	28	0	0	10	20	3	19	
4	11	5	28	3	27	-4	12	6	317	319	9	5	12	7	100	93	23	-3	13	8	42	17	42	1	1	10	244	243	4	
6	11	5	100	86	21	-2	12	6	495	503	10	7	12	7	203	176	20	-1	13	8	41	12	41	0	2	10	269	276	3	
8	11	5	118	91	23	0	12	6	178	191	12	-6	13	7	374	380	21	1	13	8	76	54	32	2	2	10	155	156	5	
-7	12	5	128	149	22	2	12	6	71	76	30	-4	13	7	330	313	14	3	13	8	166	140	19	-1	3	10	218	216	5	
-5	12	5	0	1	1	4	12	6	135	137	15	-2	13	7	432	438	9	5	13	8	96	42	50	1	3	10	279	281	6	
-3	12	5	48	53	47	6	12	6	176	165	18	0	13	7	69	58	37	-2	14	8	139	139	39	3	3	10	328	357	8	
-1	12	5	194	192	11	8	12	6	82	88	82	2	13	7	63	12	55	0	14	8	85	62	51	-2	4	10	228	241	7	
1	12	5	150	159	13	-5	13	6	229	215	14	4	13	7	71	74	40	2	14	8	63	34	63	0	4	10	295	307	8	
3	12	5	271	277	9	-3	13	6	142	150	16	6	13	7	70	68	70	0	1	9	115	105	4	2	4	10	53	76	12	
5	12	5	33	34	33	-1	13	6	477	491	8	-3	14	7	382	388	18	-1	2	9	226	248	5	4	4	10	86	106	8	
7	12	5	58	23	57	1	13	6	443	461	18	-1	14	7	140	151	24	1	2	9	1059	1112	14	-3	5	10	4	7	4	
-6	13	5	86	91	47	3	13	6	192	185	13	1	14	7	216	205	14	-2	3	9	189	200	7	-1	5	10	432	432	8	
-4	13	5	49	30	49	5	13	6	287	261	19	3	14	7	279	282	18	0	3	9	25	23	25	1	5	10	0	11	1	
-2	13	5	99	132	26	-4	14	6	95	31	94	0	0	8	938	904	66	2	3	9	0	45	1	3	5	10	151	157	6	
0	13	5	23	17	23	-2	14	6	81	79	42	1	1	8	20	28	19	-3	4	9	799	795	25	5	5	10	346	341	15	
2	13	5	227	223	11	0	14	6	263	276	11	0	2	8	340	334	7	-1	4	9	232	220	6	-4	6	10	504	507	15	
4	13	5	131	82	17	2	14	6	262	264	12	2	2	8	301	329	4	1	4	9	49	48	13	-2	6	10	91	103	9	
6	13	5	95	83	47	4	14	6	302	335	33	-1	3	8	251	250	8	3	4	9	292	304	6	0	6	10	0	29	1	
-3	14	5	31	56	31	0	1	7	467	439	7	1	3	8	281	301	7	-4	5	9	36	52	31	2	6	10	482	493	10	
-1	14	5	106	107	23	-1	2	7	803	845	24	3	3	8	202	205	6	-2	5	9	467	475	13	4	6	10	40	32	40	
1	14	5	105	95	22	-2	3	7	522	547	14	-2	4	8	147	151	4	0	5	9	811	809	19	6	6	10	12	12	11	
3	14	5	72	63	71	0	3	7	391	406	14	0	4	8	231	238	8	2	5	9	221	241	5	-5	7	10	89	125	17	
1	1	6	65	51	5	2	3	7	26	31	26	2	4	8	166	171	4	4	5	9	547	545	14	-3	7	10	0	7	1	
0	2	6	419	413	12	-3	4	7	140	140	4	4	4	8	221	215	7	-5	6	9	245	246	9	-1	7	10	236	244	8	
2	2	6	256	259	5	-1	4	7	157	137	5	-3	5	8	176	191	4	-3	6	9	242	251	7	1	7	10	338	348	8	
1	3	6	492	467	10	1	4	7	110	118	5	-1	5	8	205	204	5	-1	6	9	218	221	8	3	7	10	0	2	1	
3	3	6	259	245	6	3	4	7	148	165	4	1	5	8	204	199	6	1	6	9	243	250	6	5	7	10	395	409	16	
-2	4	6	72	65	6	-4	5	7	196	211	5	3	5	8	290	295	5	3	6	9	305	319	4	7	7	10	97	103	18	
0	4	6	552	549	12	-2	5	7	72	96	7	5	5	8	151	161	6	5	6	9	283	272	11	-6	8	10	115	136	15	
4	4	6	38	45	18	0	5	7	129	137	5	-4	6	8	400	399	12	-6	7	9	482	474	23	-4	8	10	178	181	8	
-3	5	6	323	319	6	2	5	7	555	548	11	-2	6	8	64	72	9	-4	7	9	516	508	16	-2	8	10	317	317	9	
-1	5	6	145	143	7	4	5	7	800	765	21	0	6	8	245	258	6	-2	7	9	545	535	13	0	8	10	125	143	9	
1	5	6	45	20	12	-5	6	7	144	145	6	2	6	8	89	103	8	0	7	9	198	192	8	2	8	10	43	27	43	
3	5	6	268	267	7	-3	6	7	54	64	12	4	6	8	63	63	14	2	7	9	43	47	27	4	8	10	159	164	8	
5	5	6	1458	1444	37	-1	6	7	66	76	11	6	6	8	201	191	13	4	7	9	79	88	13	6	8	10	0	30	1	
-4	6	6	689	679	12	1	6	7	390	388	7	-5	7	8	88	82	13	6	7	9	228	203	11	8	8	10	361	352	29	
-2	6	6	321	308	7	3	6	7	78	86	10	-3	7	8	27	25	26	-7	8	9	267	252	14	-7	9	10	195	217	13	
0	6	6	555	543	11	5	6	7	551	515	19	-1	7	8	246	238	8	-5	8	9	303	302	10	-5	9	10	328	340	17	
2	6	6	562	568	9	-6	7	7	57	72	23	1	7	8	103	116	8	-3	8	9	692	672	14	-3	9	10	471	463	9	
4	6	6	275	278	8	-4	7	7	321	313	7	3	7	8	0	23	1	-1	8	9	509	511	14	-1	9	10	266	257	8	
6	6	6	56	62	20	-2	7	7	351	351	9	5	7	8	126	118	8	1	8	9	362	370	8	1	9	10	413	417	7	
-5	7	6	292	303	7	0	7	7	855	866	18	7	7	8	207	191	13	3	8	9	124	146	9	3	9	10	143	159	10	
-3	7	6	185	190	6	2	7	7	268	255	4	-6	8	8	120	143	14	5	8	9	225	217	10	5	9	10	128	140	13	
-1	7	6	10	19	10	4	7	7	62	70	16	-4	8	8	39	36	39	7	8	9	51	48	50	7	9	10	376	360	29	
1	7	6	1413	1461	20	6	7	7	32	12	32	-2	8	8	127	148	10	-8	9	9	213	204	14	9	9	10	162	159	21	
3	7	6	509	504	9	-7	8	7	205	203	14	0	8	8	286	291	6	-6	9	9	408	412	21	-8	10	10	60	39	60	
5	7	6	197	181	10	-5	8	7	58	51	23	2	8	8	390	376	7	-4	9	9	44	57	44	-6	10	10	202	200	11	
-4	10	10	0	28	1	0	11	11	92	93	19	1	13	12	260	259	16	-4	6	14	52	48	39	-6	9	15	73	88	34	
-2	10	10	227	233	8	2	11	11	137	139	14	3	13	12	90	88	47	-2	6	14	142	153	7	-4	9	15	105	115	22	
0	10	10	91	114	17	4	11	11	82	68	27	0	1	13	677	677	5	0	6	14	52	33	22	-2	9	15	23	17	22	
2	10	10	86	84	18	6	11	11	121	116	22	-1	2	13	118	122	5	2	6	14	74	80	14	0	9	15	279	296	12	
4	10	10	264	271	8	-5	12	11	68	96	67	1	2	13	226	213	4	4	6	14	124	123	9	2	9	15	112	115	15	
6	10	10	153	177	16	-3	12	11	75	97	34	-2	3	13	100	111	8	6	6	14	24	58	24	4	9	15	137	156	13	
8	10	10	72	81	72	-1	12	11	196	192	12	0	3	13	124	129	6	-5	7	14	14	49	14	6	9	15	152	178	15	
-7	11	10	428	449	25	1	12	11	61	50	51	2	3	13	188	205	5	-3	7	14	103	102	12	8	9	15	103	99	26	
-5	11	10	146	162	17	3	12	11	102	96	22	-3	4	13	223	229	5	-1	7	14	294	289	9	-7	10	15	244	235	19	
-3	11	10	154	168	13	5	12	11	54	37	54	-1	4	13	234	226	6	1	7	14	140	145	9	-5	10	15	165	159	14	
-1	11	10	209	208	9	-4	13	11	0	86	1	1	4	13	831	837	17	3	7	14	0	27	1	-3	10	15	409	395	24	
1	11	10	37	34	37	-2	13	11	40	10	39	3	4	13	352	366	5	5	7	14	257	266	9	-1	10	15	119	131	15	
3	11	10	325	339	10	0	13	11	104	92	23	-4	5	13	273	274	8	7	7	14	92	98	22	1	10	15	263	275	12	
5	11	10	21	34	21	2	13	11	191	211	13	-2	5	13	826	829	25	-6	8	14	58	75	58	3	10	15	45	10	44	
7	11	10	272	265	26	4	13	11	72	70	72	0	5	13	216	215	7	-4	8	14	93	82	23	5	10	15	0	17	1	
-6	12	10	124	130	25	0	0	12	1541	1622	31	2	5	13	479	483	11	-2	8	14	59	49	26	7	10	15	408	340	44	
-4	12	10	262	275	12	1	1	12	351	361	4	4	5	13	160	171	7	0	8	14	0	42	1	-6	11	15	181	195	22	
-2	12	10	232	232	12	0	2	12	50	49	16	-5	6	13	329	328	18	2	8	14	83	84	17	-4	11	15	347	335	22	
0	12	10	131	146	16	2	2	12	312	315	4	-3	6	13	646	662	29	4	8	14	228	229	10	-2	11	15	133	145	17	
2	12	10	323	327	9	-1	3	12	175	192	5	-1	6	13	229	235	8	6	8	14	30	33	29	0	11	15	164	189	13	
4	12	10	51	51	51	1	3	12	434	426	7	1	6	13	23	12	23	8	8	14	69	88	45	2	11	15	79	86	30	
6	12	10	127	118	23	3	3	12	649	680	10	3	6	13	199	204	6	-7	9	14	149	151	17	4	11	15	0	19	1	
-5	13	10	55	12	55	-2	4	12	126	141	6	5	6	13	151	170	8	-5	9	14	246	248	26	6	11	15	81	123	81	
-3	13	10	63	63	62	0	4	12	161	170	5	-6	7	13	778	748	51	-3	9	14	82	80	21	-3	12	15	37	65	37	
-1	13	10	380	392	13	2	4	12	1417	1508	25	-4	7	13	261	256	15	-1	9	14	0	8	1	-1	12	15	112	131	22	
1	13	10	146	153	18	4	4	12	200	222	7	-2	7	13	167	174	8	1	9	14	22	51	21	1	12	15	128	130	20	
3	13	10	261	250	11	-3	5	12	302	325	10	0	7	13	282	287	9	3	9	14	50	44	50	3	12	15	385	413	22	
5	13	10	214	241	25	-1	5	12	455	452	11	2	7	13	151	155	7	5	9	14	123	140	15	0	0	16	525	498	10	
0	1	11	48	35	11	1	5	12	112	113	7	4	7	13	44	58	43	7	9	14	28	26	27	1	1	16	259	259	5	
-1	2	11	76	96	7	3	5	12	440	457	10	6	7	13	369	371	14	9	9	14	0	5	1	0	2	16	261	267	6	
1	2	11	36	19	20	5	5	12	35	38	35	-7	8	13	592	561	48	-8	10	14	110	65	35	2	2	16	63	57	15	
-2	3	11	66	65	11	-4	6	12	115	121	10	-5	8	13	42	57	41	-6	10	14	93	110	37	-1	3	16	66	69	14	
0	3	11	136	146	5	-2	6	12	1002	1040	35	-3	8	13	192	190	8	-4	10	14	34	27	33	1	3	16	21	45	21	
2	3	11	482	477	8	0	6	12	529	523	17	-1	8	13	210	210	8	-2	10	14	72	71	26	3	3	16	311	319	5	
-3	4	11	298	298	9	2	6	12	274	278	5	1	8	13	400	409	10	0	10	14	75	81	24	-2	4	16	470	455	7	
-1	4	11	27	36	27	4	6	12	100	99	11	3	8	13	780	776	21	2	10	14	74	87	27	0	4	16	360	360	6	
1	4	11	111	130	7	6	6	12	123	142	12	5	8	13	138	132	12	4	10	14	81	84	25	2	4	16	110	121	9	
3	4	11	32	44	31	-5	7	12	71	82	23	7	8	13	44	46	43	6	10	14	92	77	32	4	4	16	100	115	12	
-4	5	11	344	348	11	-3	7	12	112	123	11	-8	9	13	317	288	30	8	10	14	161	119	55	-3	5	16	117	118	10	
-2	5	11	59	74	13	-1	7	12	444	452	13	-6	9	13	49	58	49	-5	11	14	103	41	24	-1	5	16	509	495	15	
0	5	11	279	279	9	1	7	12	47	51	28	-4	9	13	81	85	26	-3	11	14	60	48	59	1	5	16	0	7	1	
2	5	11	32	55	31	3	7	12	159	180	7	-2	9	13	160	172	10	-1	11	14	32	20	32	3	5	16	381	383	7	
4	5	11	177	184	6	5	7	12	667	653	22	0	9	13	404	401	8	1	11	14	303	306	12	5	5	16	16	31	15	
-5	6	11	130	127	13	7	7	12	55	60	55	2	9	13	332	334	11	3	11	14	55	45	55	-4	6	16	315	320	18	
-3	6	11	45	53	24	-6	8	12	649	626	43	4	9	13	99	93	17	5	11	14	52	85	52	-2	6	16	122	133	10	
-1	6	11	212	219	7	-4	8	12	48	54	48	6	9	13	126	152	17	-4	12	14	41	73	41	0	6	16	105	118	12	
1	6	11	215	215	7	-2	8	12	17	5	16	8	9	13	99	97	26	-2	12	14	31	11	30	2	6	16	678	666	15	
3	6	11	181	193	6	0	8	12	86	103	13	-7	10	13	41	46	40	0	12	14	0	9	1	4	6	16	147	161	9	
5	6	11	79	87	14	2	8	12	48	8	35	-5	10	13	252	243	20	2	12	14	15	58	14	6	6	16	14	49	13	
-6	7	11	39	46	39	4	8	12	1145	1134	30	-3	10	13	238	238	12	4	12	14	57	31	56	-5	7	16	61	91	44	
-4	7	11	133	133	10	6	8	12	233	242	17	-1	10	13	618	618	18	0	1	15	405	393	4	-3	7	16	96	102	16	
-2	7	11	0	11	1	8	8	12	78	98	31	1	10	13	347	359	9	-1	2	15	52	64	18	-1	7	16	151	159	12	
0	7	11	456	441	16	-7	9	12	27	34	26	3	10	13	33	14	33	1	2	15	709	708	5	1	7	16	17	9	16	
2	7	11	92	96	10	-5	9	12	466	463	29	5	10	13	105	116	21	-2	3	15	0	20	1	3	7	16	40	30	39	
4	7	11	278	277	7	-3	9	12	439	426	16	7	10	13	127	156	24	0	3	15	66	73	12	5	7	16	360	376	10	
6	7	11	231	235	14	-1	9	12	287	287	11	-6	11	13	51	11	51	2	3	15	241	239	5	7	7	16	222	223	11	
-7	8	11	76	73	28	1	9	12	499	511	10	-4	11	13	426	414	23	-3	4	15	547	547	12	-6	8	16	350	334	24	
-5	8	11	114	126	14	3	9	12	67	87	24	-2	11	13	232	235	10	-1	4	15	82	72	11	-4	8	16	0	18	1	
-3	8	11	94	104	15	5	9	12	45	48	44	0	11	13	35	19	35	1	4	15	285	300	5	-2	8	16	523	498	23	
-1	8	11	109	123	12	7	9	12	263	218	33	2	11	13	14	32	14	3	4	15	69	83	15	0	8	16	93	86	16	
1	8	11	5	30	5	9	9	12	86	88	33	4	11	13	309	290	10	-4	5	15	79	91	15	2	8	16	44	39	44	
3	8	11	103	125	12	-8	10	12	94	87	28	6	11	13	113	104	32	-2	5	15	566	564	13	4	8	16	152	159	11	
5	8	11	247	249	12	-6	10	12	442	423	34	-5	12	13	535	534	21	0	5	15	543	538	11	6	8	16	116	119	18	
7	8	11	31	13	30	-4	10	12	87	123	22	-3	12	13	95	98	33	2	5	15	180	179	7	8	8	16	408	379	32	
-8	9	11	28	31	28	-2	10	12	53	70	53	-1	12	13	66	59	46	4	5	15	301	317	8	-7	9	16	175	176	15	
-6	9	11	276	261	21	0	10	12	588	600	14	1	12	13	249	238	13	-5	6	15	261	255	15	-5	9	16	248	239	21	
-4	9	11	33	20	32	2	10	12	305	294	7	3	12	13	380	384	15	-3	6	15	83	87	16	-3	9	16	396	370	33	
-2	9	11	73	70	20	4	10	12	87	81	21	5	12	13	632	613	54	-1	6	15	58	63	18	-1	9	16	298	282	24	
0	9	11	61	80	27	6	10	12	205	195	16	-2	13	13	107	106	42	1	6	15	131	140	9	1	9	16	500	519	18	
2	9	11	191	203	8	8	10	12	521	515	26	0	13	13	352	319	15	3	6	15	140	158	9	3	9	16	87	87	22	
4	9	11	187	182	9	-7	11	12	362	326	47	2	13	13	77	39	77	5	6	15	343	359	12	5	9	16	124	122	17	
6	9	11	74	91	26	-5	11	12	266	269	15	0	0	14	688	648	16	-6	7	15	583	570	34	7	9	16	95	122	28	
8	9	11	179	162	20	-3	11	12	141	155	13	1	1	14	166	175	5	-4	7	15	299	303	18	-6	10	16	139	112	21	
-9	10	11	90	83	58	-1	11	12	238	230	9	0	2	14	158	163	5	-2	7	15	68	71	21	-4	10	16	64	7	49	
-7	10	11	145	151	16	1	11	12	497	503	10	2	2	14	14	22	14	0	7	15	22	26	21	-2	10	16	132	139	17	
-5	10	11	44	49	44	3	11	12	198	203	12	-1	3	14	240	232	5	2	7	15	120	135	11	0	10	16	321	340	13	
-3	10	11	76	97	22	5	11	12	90	84	29	1	3	14	86	91	8	4	7	15	112	118	11	2	10	16	77	76	27	
-1	10	11	66	74	29	7	11	12	420	431	27	3	3	14	325	336	5	6	7	15	117	123	15	4	10	16	311	335	9	
1	10	11	72	49	23	-6	12	12	0	23	1	-2	4	14	18	16	18	-7	8	15	32	57	31	6	10	16	292	291	15	
3	10	11	54	46	46	-4	12	12	236	250	17	0	4	14	114	116	7	-5	8	15	48	56	47	-5	11	16	192	188	20	
5	10	11	56	12	56	-2	12	12	342	345	10	2	4	14	394	393	6	-3	8	15	533	506	36	-3	11	16	97	124	25	
7	10	11	76	25	51	0	12	12	91	81	24	4	4	14	50	50	23	-1	8	15	348	336	12	-1	11	16	66	71	46	
9	10	11	149	153	35	2	12	12	30	5	29	-3	5	14	46	43	32	1	8	15	375	388	10	1	11	16	47	88	46	
-8	11	11	88	82	46	4	12	12	241	251	11	-1	5	14	66	87	13	3	8	15	296	304	7	3	11	16	85	108	29	
-6	11	11	6	40	6	6	12	12	372	394	23	1	5	14	153	155	6	5	8	15	42	52	41	5	11	16	145	149	31	
-4	11	11	85	62	24	-3	13	12	487	482	38	3	5	14	108	121	10	7	8	15	7	41	7	-2	12	16	237	233	27	
-2	11	11	70	71	27	-1	13	12	368	355	13	5	5	14	117	135	10	-8	9	15	77	85	39	0	12	16	206	224	18	
2	12	16	245	228	18	-5	7	18	42	84	42	1	10	19	220	219	13	-3	8	21	318	298	28	3	8	23	0	57	1	
0	1	17	134	141	9	-3	7	18	34	9	34	3	10	19	99	93	25	-1	8	21	168	164	15	0	0	24	930	911	29	
-1	2	17	50	67	33	-1	7	18	93	97	18	0	11	19	335	334	14	1	8	21	0	46	1	1	1	24	238	243	11	
1	2	17	447	456	5	1	7	18	705	700	23	0	0	20	197	185	17	3	8	21	170	195	13	0	2	24	61	82	44	
-2	3	17	102	108	10	3	7	18	217	230	8	1	1	20	81	89	18	5	8	21	64	38	63	2	2	24	15	53	14	
0	3	17	66	61	17	5	7	18	167	173	12	0	2	20	82	93	20	-4	9	21	103	86	102	-1	3	24	71	44	44	
2	3	17	37	10	37	7	7	18	0	26	1	2	2	20	249	245	9	-2	9	21	90	68	34	1	3	24	234	216	11	
-3	4	17	340	337	5	-6	8	18	219	204	14	-1	3	20	594	568	8	0	9	21	139	135	24	3	3	24	431	439	9	
-1	4	17	277	267	7	-4	8	18	89	85	24	1	3	20	336	330	8	2	9	21	125	147	20	-2	4	24	151	173	15	
1	4	17	22	18	21	-2	8	18	248	229	23	3	3	20	160	157	11	4	9	21	93	115	56	0	4	24	53	34	53	
3	4	17	119	127	10	0	8	18	116	116	15	-2	4	20	121	119	14	-1	10	21	34	50	34	2	4	24	519	519	7	
-4	5	17	59	60	27	2	8	18	482	482	18	0	4	20	249	235	8	1	10	21	109	75	37	4	4	24	0	21	1	
-2	5	17	273	280	11	4	8	18	78	84	23	2	4	20	260	245	8	0	0	22	64	92	63	-3	5	24	113	89	25	
0	5	17	339	345	10	6	8	18	17	28	17	4	4	20	79	85	25	1	1	22	214	214	9	-1	5	24	118	123	23	
2	5	17	4	15	4	8	8	18	508	439	42	-3	5	20	92	115	18	0	2	22	110	113	16	1	5	24	218	204	11	
4	5	17	209	206	7	-7	9	18	37	106	36	-1	5	20	92	95	18	2	2	22	0	8	1	3	5	24	159	137	15	
-5	6	17	43	37	42	-5	9	18	51	55	50	1	5	20	109	111	17	-1	3	22	25	13	24	5	5	24	144	142	21	
-3	6	17	97	100	15	-3	9	18	514	491	38	3	5	20	68	70	29	1	3	22	221	222	11	-4	6	24	129	144	24	
-1	6	17	106	110	13	-1	9	18	97	116	22	5	5	20	558	539	23	3	3	22	318	311	10	-2	6	24	402	380	16	
1	6	17	146	146	9	1	9	18	0	21	1	-4	6	20	85	68	25	-2	4	22	72	68	30	0	6	24	68	19	51	
3	6	17	72	73	18	3	9	18	100	108	20	-2	6	20	0	45	1	0	4	22	155	155	12	2	6	24	86	76	28	
5	6	17	126	136	12	5	9	18	86	118	29	0	6	20	372	374	11	2	4	22	89	66	21	4	6	24	18	4	18	
-6	7	17	267	265	15	7	9	18	388	360	15	2	6	20	282	281	7	4	4	22	19	6	18	-3	7	24	131	141	45	
-4	7	17	280	269	16	-4	10	18	81	97	38	4	6	20	17	59	17	-3	5	22	0	15	1	-1	7	24	554	488	45	
-2	7	17	226	221	12	-2	10	18	527	489	55	6	6	20	42	46	41	-1	5	22	325	323	13	1	7	24	137	123	20	
0	7	17	23	23	23	0	10	18	208	206	13	-5	7	20	63	75	62	1	5	22	73	68	29	3	7	24	60	25	60	
2	7	17	52	50	40	2	10	18	165	174	14	-3	7	20	39	19	39	3	5	22	196	194	12	0	8	24	110	63	53	
4	7	17	43	15	42	4	10	18	434	440	16	-1	7	20	7	57	6	5	5	22	103	103	27	0	1	25	222	233	11	
6	7	17	56	29	55	-3	11	18	85	48	84	1	7	20	560	534	16	-4	6	22	363	346	19	-1	2	25	0	19	1	
-7	8	17	74	77	38	-1	11	18	72	38	45	3	7	20	105	125	17	-2	6	22	0	16	1	1	2	25	33	36	33	
-5	8	17	166	157	16	1	11	18	48	26	47	5	7	20	122	117	18	0	6	22	134	136	16	-2	3	25	198	197	13	
-3	8	17	390	368	29	3	11	18	353	363	16	7	7	20	95	108	33	2	6	22	348	320	12	0	3	25	87	93	27	
-1	8	17	289	275	18	0	1	19	94	87	14	-6	8	20	23	11	23	4	6	22	80	84	31	2	3	25	283	279	10	
1	8	17	74	87	21	-1	2	19	737	707	8	-4	8	20	136	135	18	6	6	22	91	62	34	-3	4	25	68	47	54	
3	8	17	29	68	28	1	2	19	187	184	9	-2	8	20	96	88	33	-5	7	22	149	146	20	-1	4	25	421	416	16	
5	8	17	129	125	16	-2	3	19	642	631	8	0	8	20	131	130	15	-3	7	22	99	95	29	1	4	25	624	588	12	
7	8	17	147	134	17	0	3	19	320	325	7	2	8	20	216	220	10	-1	7	22	266	248	20	3	4	25	150	167	18	
-6	9	17	200	197	24	2	3	19	225	229	8	4	8	20	294	293	16	1	7	22	112	103	24	-4	5	25	59	71	59	
-4	9	17	0	24	1	-3	4	19	118	129	13	6	8	20	79	60	43	3	7	22	0	17	1	-2	5	25	383	358	10	
-2	9	17	146	155	16	-1	4	19	336	334	6	-5	9	20	89	62	88	5	7	22	384	339	22	0	5	25	72	51	50	
0	9	17	90	71	22	1	4	19	3	26	2	-3	9	20	208	172	18	-4	8	22	224	226	22	2	5	25	158	149	16	
2	9	17	40	49	39	3	4	19	77	78	22	-1	9	20	81	70	31	-2	8	22	240	226	20	4	5	25	72	27	59	
4	9	17	269	275	11	-4	5	19	2	35	2	1	9	20	100	95	24	0	8	22	24	39	23	-3	6	25	455	467	17	
6	9	17	31	69	30	-2	5	19	124	135	12	3	9	20	35	39	34	2	8	22	51	32	50	-1	6	25	160	152	22	
-5	10	17	111	104	22	0	5	19	107	112	13	5	9	20	204	210	15	4	8	22	151	183	18	1	6	25	67	36	66	
-3	10	17	35	55	35	2	5	19	229	228	8	-2	10	20	190	178	25	-1	9	22	159	172	39	3	6	25	103	112	30	
-1	10	17	51	32	50	4	5	19	803	785	24	0	10	20	0	12	1	1	9	22	383	368	21	0	7	25	122	44	46	
1	10	17	74	91	33	-5	6	19	57	45	56	2	10	20	155	157	17	3	9	22	193	196	24	0	0	26	971	926	12	
3	10	17	64	54	42	-3	6	19	163	178	12	0	1	21	24	27	23	0	1	23	108	115	16	1	1	26	0	14	1	
5	10	17	87	103	35	-1	6	19	52	48	51	-1	2	21	254	240	8	-1	2	23	0	32	1	0	2	26	53	58	53	
-4	11	17	129	105	31	1	6	19	341	340	7	1	2	21	230	238	10	1	2	23	555	536	7	2	2	26	103	78	24	
-2	11	17	125	153	22	3	6	19	198	201	9	-2	3	21	198	178	10	-2	3	23	41	34	41	-1	3	26	211	200	14	
0	11	17	247	254	16	5	6	19	489	486	19	0	3	21	254	246	8	0	3	23	109	126	17	1	3	26	228	235	12	
2	11	17	82	93	29	-6	7	19	167	165	17	2	3	21	316	289	8	2	3	23	20	49	19	3	3	26	207	220	15	
4	11	17	92	101	45	-4	7	19	263	247	13	-3	4	21	357	363	10	-3	4	23	459	446	15	-2	4	26	78	73	40	
0	0	18	113	111	23	-2	7	19	287	263	22	-1	4	21	109	104	16	-1	4	23	21	20	20	0	4	26	63	63	63	
1	1	18	40	57	39	0	7	19	532	519	21	1	4	21	161	162	13	1	4	23	44	50	44	4	4	26	95	93	49	
0	2	18	393	400	6	2	7	19	325	316	10	3	4	21	94	109	21	3	4	23	27	51	27	-3	5	26	309	304	21	
2	2	18	41	80	41	4	7	19	146	146	12	-4	5	21	352	353	13	-4	5	23	156	149	18	-1	5	26	0	20	1	
-1	3	18	1007	981	12	6	7	19	228	219	12	-2	5	21	143	159	14	-2	5	23	239	232	14	1	5	26	78	84	38	
1	3	18	163	168	8	-7	8	19	115	114	27	0	5	21	359	359	13	0	5	23	461	445	18	3	5	26	0	24	1	
3	3	18	205	212	7	-5	8	19	18	6	18	2	5	21	173	175	11	2	5	23	40	13	40	0	6	26	252	212	40	
-2	4	18	167	174	9	-3	8	19	628	572	49	4	5	21	335	328	9	4	5	23	146	157	16	0	1	27	70	104	56	
0	4	18	531	503	10	-1	8	19	245	236	12	-5	6	21	218	208	11	-5	6	23	256	240	13	-1	2	27	53	27	53	
2	4	18	98	96	15	1	8	19	131	138	14	-3	6	21	0	16	1	-3	6	23	89	84	33	1	2	27	105	123	25	
4	4	18	141	151	10	3	8	19	20	25	19	-1	6	21	8	9	7	-1	6	23	24	44	23	-2	3	27	0	23	1	
-3	5	18	92	99	15	5	8	19	55	62	54	1	6	21	333	312	11	1	6	23	55	5	55	0	3	27	27	36	27	
-1	5	18	33	38	32	7	8	19	118	81	28	3	6	21	306	295	12	3	6	23	185	169	13	2	3	27	301	291	11	
1	5	18	118	115	11	-6	9	19	279	272	14	5	6	21	14	10	13	5	6	23	307	281	13	-1	4	27	49	51	49	
3	5	18	57	54	25	-4	9	19	616	544	53	-6	7	21	228	220	16	-4	7	23	307	267	26	1	4	27	142	146	23	
5	5	18	685	686	24	-2	9	19	164	166	31	-4	7	21	201	207	23	-2	7	23	145	146	21	0	0	28	205	162	35	
-4	6	18	547	531	26	0	9	19	97	90	23	-2	7	21	222	208	16	0	7	23	34	9	33	1	1	28	0	52	1	
-2	6	18	226	226	12	2	9	19	187	179	14	0	7	21	451	437	20	2	7	23	177	177	16	0	2	28	122	153	37	
0	6	18	124	117	12	4	9	19	363	389	11	2	7	21	85	67	24	4	7	23	54	16	53	2	2	28	168	158	25	
2	6	18	449	444	13	6	9	19	737	664	32	4	7	21	98	109	23	-3	8	23	238	267	45							
4	6	18	22	29	22	-3	10	19	75	30	71	6	7	21	146	158	22	-1	8	23	328	281	25							
6	6	18	231	222	11	-1	10	19	147	152	16	-5	8	21	87	78	35	1	8	23	340	310	19							
																														
																														
																														
